# Uncovering the molecular signature underlying the light intensity-dependent root development in *Arabidopsis thaliana*

**DOI:** 10.1186/s12864-019-5933-5

**Published:** 2019-07-20

**Authors:** Sony Kumari, Sandeep Yadav, Debadutta Patra, Sharmila Singh, Ananda K. Sarkar, Kishore C. S. Panigrahi

**Affiliations:** 1 School of Biological Sciences, National Institute of Science Education and Research (NISER), Homi Bhabha National Institute (HBNI), P.O. Bhimpur- Padanpur, Via Jatni, Dist. Khurda, Odisha 752050 India; 2 National Institute of Plant Genome Research (NIPGR), Jawaharlal Nehru University Campus, Aruna Asaf Ali Marg, New Delhi, Delhi 110067 India

**Keywords:** Root, Light signaling, Intensity, Gene expression, Auxin, Hormone

## Abstract

**Background:**

Root morphology is known to be affected by light quality, quantity and direction. Light signal is perceived at the shoot, translocated to roots through vasculature and further modulates the root development. Photoreceptors are differentially expressed in both shoot and root cells. The light irradiation to the root affects shoot morphology as well as whole plant development. The current work aims to understand the white light intensity dependent changes in root patterning and correlate that with the global gene expression profile.

**Results:**

Different fluence of white light (WL) regulate overall root development via modulating the expression of a specific set of genes. Phytochrome A deficient *Arabidopsis thaliana* (*phyA-211*) showed shorter primary root compared to phytochrome B deficient (*phyB-9*) and wild type (WT) seedlings at a lower light intensity. However, at higher intensity, both mutants showed shorter primary root in comparison to WT. The lateral root number was observed to be lowest in *phyA-211* at intensities of 38 and 75 μmol m ^− ^^2^ s ^− ^^1^. The number of adventitious roots was significantly lower in *phyA-211* as compared to WT and *phyB-9* under all light intensities tested. With the root phenotypic data, microarray was performed for four different intensities of WL light in WT. Here, we identified ~ 5243 differentially expressed genes (DEGs) under all light intensities. Gene ontology-based analysis indicated that different intensities of WL predominantly affect a subset of genes having catalytic activity and localized to the cytoplasm and membrane. Furthermore, when root is irradiated with different intensities of WL, several key genes involved in hormone, light signaling and clock-regulated pathways are differentially expressed.

**Conclusion:**

Using genome wide microarray-based approach, we have identified candidate genes in *Arabidopsis* root that responded to the changes in light intensities. Alteration in expression of genes such as *PIF4*, *COL9*, *EPR1, CIP1, ARF18, ARR6, SAUR9, TOC1* etc. which are involved in light, hormone and clock pathway was validated by qRT-PCR. This indicates their potential role in light intensity mediated root development.

**Electronic supplementary material:**

The online version of this article (10.1186/s12864-019-5933-5) contains supplementary material, which is available to authorized users.

## Background

Light is an essential parameter for the optimal growth and survival of plants. The quality, quantity, direction and duration of light are important factors required for various aspects of plant development [1]. Root development comprises of different aspects such as primary root elongation, lateral root elongation, lateral root branching, root geotropism, root hair formation etc. Root patterning beneath the soil plays a crucial role in penetration, anchorage and gravitropism leading to absorption of water and nutrient. To perceive light, plants have evolved with many canonical photoreceptors such as phytochromes (PHYs), cryptochromes (CRYs), phototropins (PHOTs) and UVB-resistance 8 (UVR8) [2, 3]. Light regulates the patterning of shoot as well as root system [4]. It has been shown to regulate all the aforesaid aspects of root development at different stages of plant life-cycle [5–8]. Light signal from shoot can translocate through phloem to root and alters light-mediated responses [9]. Although, roots grown beneath the soil generally don’t experience direct light, still few parts of the root are exposed to some amount of light seeping through the cracks, pores of the soil and affects the overall root development. Light percolates through the soil and reaches the root which leads to the production of reactive oxygen species (ROS) and promotes root growth when present in an optimal level. Ha *et.al*., 2018 has shown the correlation of photoreceptors with ROS-mediated root growth. The shoot localized PHYs have been reported to mediate this response through ROS accumulation in roots. A shoot-localized abscisic acid (ABA) signaling component is also shown to be involved in PHYB-mediated primary root elongation. It has been shown that when roots are exposed to light, shoot PHYs induce ABA biosynthesis and signaling mediator which further promote primary root growth. PHY controls the translocation of ABA signals from shoot to root which increase the expression of *ABA INSENSITIVE 5* (*ABI5*) gene, which activates *PEROXIDASE 1* (*PER1*) in the root. *ABI5* encodes a basic leucine-rich zipper transcription factor while *PER1* encodes a peroxidase that detoxifies ROS. When root is exposed to light, activation of *PER1* leads to detoxification of ROS and maintains its level, which promotes root growth. This showed that PHYB promotes primary root growth through regulating *ABI5* and *PER1* activity and ROS accumulation [10]. *ELONGATED HYPOCOTYL 5* (*HY5*), one of the major transcription factors, downstream to PHYs has been shown to be a mobile signal. It has been documented that, *HY5* is activated in shoot, translocate to roots and regulates the root architecture [11]. Intrinsic component such as phytohormones are also involved in regulating root patterning. Auxin is one of the important phytohormones that plays a major role in root development. *HY5* has also been shown to be a mediator of PHY and auxin signaling. Along with phytohormones, nutrients such as sugar also plays an important role in root development. The cross-talk of sugar and phytohormone such as auxin has been shown to modulate the root growth and development [12]. MEDIATOR (MED) complex is one of the most important candidate that couples sugar and auxin signalling pathways in root development. In support of this, *MED12* and *MED13* genes have been shown to promote primary root length, root hair number and root hair length by enhancing the cell elongation, cell division and auxin response. Addition of sucrose compliments the root defect in *med12* and *med13* mutants. Thus, *MED12* and *MED13* are the important candidate genes which link auxin signaling and nutritional status of the root [13]. On the contrary, *med18* mutant has been reported to show shorter primary root, lesser number of lateral roots with longer and denser root hairs. The alteration of root architecture in *med18* mutant is because of altered auxin response and its distribution in primary root. Although in natural condition, roots grow under relative darkness still there is always some communication and signal translocation between shoot to root, historically known as light piping. In *med18* mutant, it has been shown that shoot perceives the light and causes the death of root meristem cells. However, cell death at root meristem occurred irrespective of direct light exposure to the roots, which suggested a long-distance communication between shoot and root is plausible. This indicated that root growth is affected in similar fashion irrespective of its light irradiation [14]. There are very few available reports which document the light and hormone cross-talk in root development [15–18]. The light quality and quantity both affect the root patterning [19]. Although, the effect of light intensity on regulation of root development has not been well investigated at molecular level and needs a systematic study.

*Nicotiana tabacum* plants grown under variable intensity of WL for different durations, showed altered root growth, leaf biomass, sugar content and chlorophyll level*.* It has been reported that, plants constantly grown either for 14 or 18 days under 60 μmol m^− 2^ s^− 1^ (condition A) light, they have lesser fresh weight of root and shoot as compared to plants grown under 300 μmol m^− 2^ s^− 1^ (condition B) light. However, plants grown for first 14 days under 60 μmol m^− 2^ s^− 1^ light and then subsequent 4 days under 300 μmol m^− 2^ s^− 1^ light (condition C), after a total of 18 days of light treatment, the fresh weight of root and shoot were observed to be intermediate of that of continuous 18 days under 60 μmol m^− 2^ s^− 1^ and 300 μmol m^− 2^ s^− 1^ light intensity. This suggested an additive effect of light fluence and duration. Further, glucose, fructose and sucrose levels were observed to be highest in plants grown under condition C compared to condition A and B. Then, it was concluded that the promotion of root growth under higher light intensity is due to enhanced carbohydrate transport from shoot to root and independent of sugar content [20]. In tomato, *Solanum lycopersicum* cyclophilins (*SlCyp1*) are peptidyl-prolyl cis/trans isomerases which play an important role in plant development. It is transported from shoot to root as a phloem mobile signal. The trafficking of *SlCyp1* is enhanced with increasing light intensity, leading to profound root growth. [21]. It has been reported that, in gymnosperms such as *Pinus sylvestris L*. (Scots pine), plants grown in the presence of different intensities of red (R) and far-red (FR) light (1, 10, 25 and 100 μmol m^− 2^ s^− 1^) showed variation in root growth. Irradiation of R light had no significant effect on root length whereas low intensity of FR light (1 and 10 μmol m^− 2^ s^− 1^) has resulted shorter root in comparison to the seedlings grown under complete darkness. However, under highest intensity of FR light, root length was significantly higher as compared to other light conditions. Therefore, it can be concluded that equal intensity of monochromatic light didn’t show similar type of effect on plant development [22]. In a recent report, *Arabidopsis thaliana* seedlings grown in two different experimental setups, in one the roots were directly exposed to WL while in another, root was covered and the shoot was exposed to light (D-root system). In this report, it has been shown that the spatial expression of the photoreceptor genes such as *UVR8*, *CRY1*, *CRY2*, *PHOT1*, *PHOT2*, *PHYA* and *PHYB* varied in different segments of roots under the above mentioned experimental setups [23]. Although light intensity has been shown to influence the root development, how it correlates with the gene expression, leading to root patterning needs to be examined in detail. In the present work whole seedlings were grown under 38, 75, 112 and 150 μmol m^− 2^ s^− 1^ intensities of WL with 16 h light and 8 h dark. The root patterning was analysed in WT and *PHY* mutants. Using microarray-based approach, we analysed the variation in the global transcription profile of the root tissue of WT seedlings. Further, we evaluated the expression pattern of various genes that play important role in light signaling, hormone signaling and clock-regulated pathways.

## Results

### Phytochrome mutants showed variation in root patterning under different intensities of white light

**Fig. 1:**
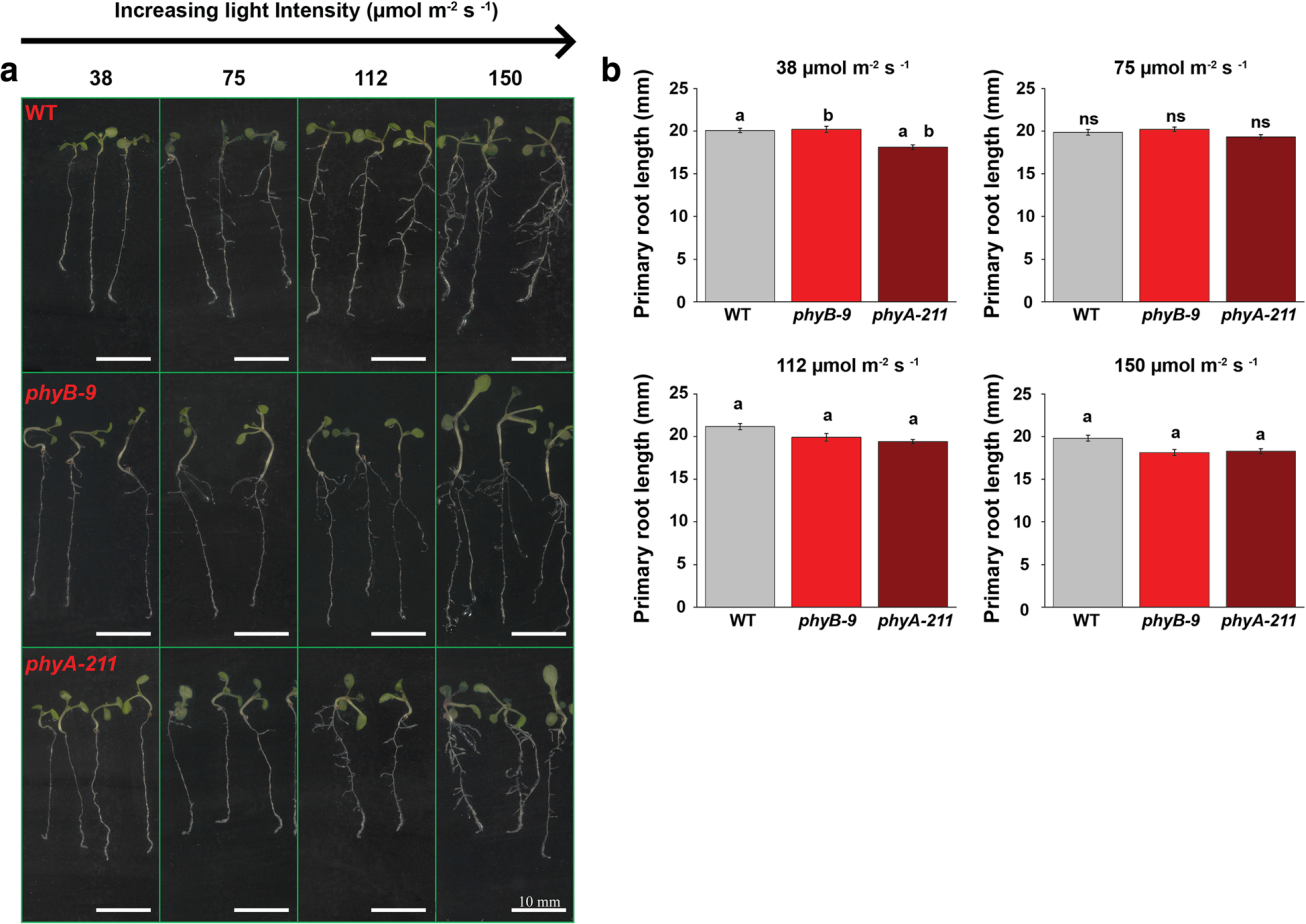
Light intensity dependent root growth varies in phytochrome mutants. **a** Phenotypic differences in root architecture. **b** Primary root length (mm) for WT, *phyB-9* and *phyA-211* under four different WL intensities of 38, 75, 112 and 150 μmol m ^−^ ^2^ s^ − ^^1^. The analysis was done in 6-days old seedlings. Error bars represent SE. The means were compared using Tukey test with *p* ≤ 0.05. Same letters denote statistical significance. There were five technical replicates, each replicate consists of 20 seedlings. Scale bar = 10 mm

**Fig. 2:**
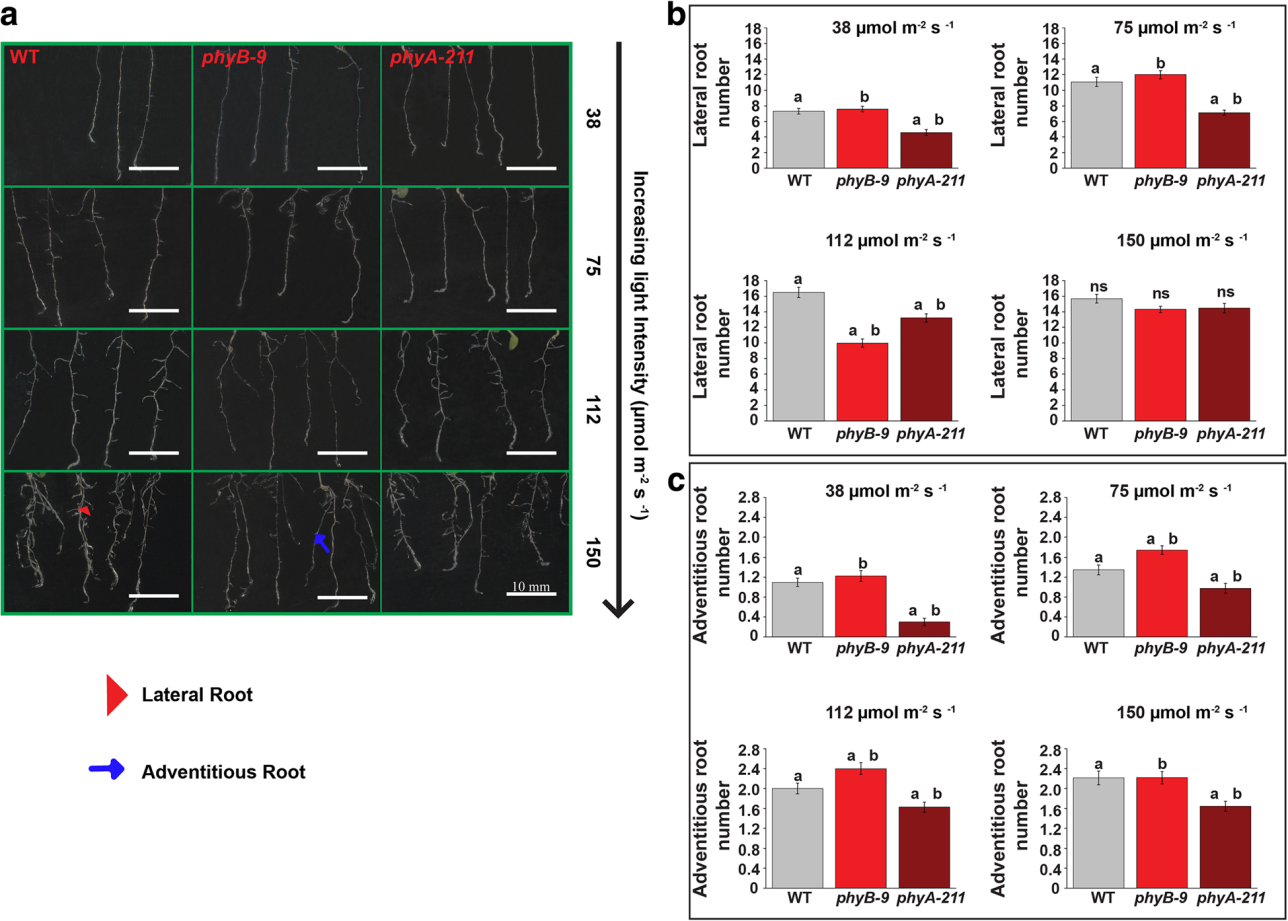
Phytochrome mutants show variability in light intensity dependent lateral and adventitious root growth**. a** Lateral and adventitious root architectural differences. The lateral and adventitious root have been indicated by arrowheads and arrows respectively. **b** Phytochrome mutants show variation in lateral root number. **c** Adventitious root number varies among the genotypes under all four WL intensity. The analysis has been done in 6-days old seedlings. Error bars represent SE. The means were compared using Tukey test with *p* ≤ 0.05. Same letters denote statistical significance There were three technical replicates, each replicate consists of 20 seedlings. Scale bar is 10 mm

*Arabidopsis* seedlings, WT, *phyB-9* and *phyA-211* showed variable root growth responses under different light intensities of 38, 75, 112 and 150 μmol m ^−^ ^2^ s ^− ^^1^ WL (Fig. 1a). Primary root length in 6-days old seedlings was found to be slightly shorter in case of *phyA-211* as compared to *phyB-9* and WT under 38 μmol m^ − ^^2^ s ^− ^^1^ light intensity. Under 150 and 112 μmol m ^− ^^2^ s ^−^ ^1^ light, the primary root length was observed to be marginally shorter in both the *PHY* mutants as compared to WT (Fig. 1b). It was also observed that lateral and adventitious root growth varied in *PHY* mutants under different light intensity in comparison to WT (Fig. 2a). The zoomed image for qualitative details of lateral root growth has been shown in Additional file 1: Figure S1. When light intensity increased from 38 to 150 μmol m ^− ^^2^ s^ − ^^1^, the number of lateral roots approximately increased by two times in case of WT, *phyB-9* and three times in *phyA-211*. However, under 112 μmol m ^− ^^2^ s ^− ^^1^ light, *phyB-9* has significantly lesser number of lateral root and about 16.7 and 30.3% reduction were observed in comparison to 75 and 150 μmol m ^− ^^2^ s ^− ^^1^ light intensity respectively. The lateral root number in *phyA-211* decreased by 12.5 and 35.7% as compared to WT under 38 μmol m^ −^ ^2^ s ^− ^^1^ and 75 μmol m ^− ^^2^ s ^− ^^1^ light respectively (Fig. 2b). Interestingly, under 112 μmol m ^− ^^2^ s ^− ^^1^ light, the lateral root number in case of *phyB-9* was reduced by 39.5 and 24.5% in comparison to WT and *phyA-211* respectively*.* At higher intensity of 150 μmol m ^− ^^2^ s ^− ^^1^, no significant difference was found in lateral root number as all the genotypes showed ~ 14–16 rootlets (Fig. 2b).

The number of adventitious roots was shown to increase by increasing light intensity till 75 μmol m ^− ^^2^ s ^− ^^1^ and apparently saturates in between 112 μmol m ^− ^^2^ s ^− ^^1^ and 150 μmol m ^− ^^2^ s ^− ^^1^. It has been observed that the adventitious root number was least in case of *phyA-211* as compared to WT and *phyB-9* under all light intensities (Fig. 2c). The adventitious root number in case of *phyA-211* was least under 38 μmol m ^− ^^2^ s ^− ^^1^ as compared to other light intensities. The microscope images of lateral root growth for qualitative visualization in 6-days old seedling have been shown in Additional file 2: Figure S2.

### Identification of differentially expressed genes under four different intensities of white light in WT root

**Fig. 3:**
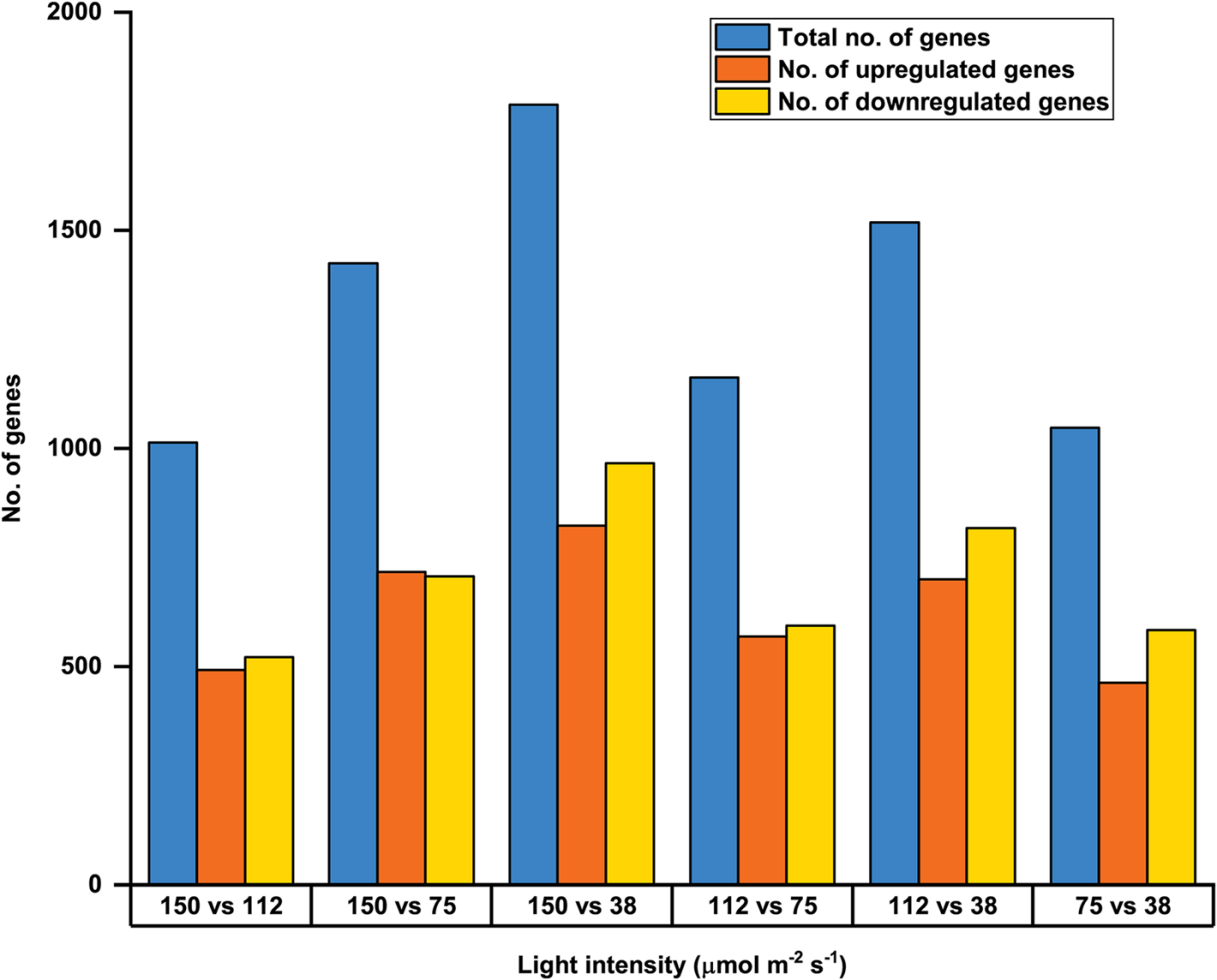
DEGs found under different comparative light intensities from microarray. Total number of DEGs, number of upregulated and downregulated DEGs under comparative light intensity of 150 vs 112, 150 vs 75, 150 vs 38, 112 vs 75, 112 vs 38 and 75 vs 38 μmol m ^− ^^2^ s ^− ^^1^ obtained from 5-days old WT root microarray analysis

In all the genotypes root architectural differences were observed to follow a pattern when grown under different intensities of WL. With this information, microarray was carried out with root samples of 5-days old WT seedlings grown under four different WL intensities (mentioned earlier). Differential expression analysis was performed to identify the differentially expressed genes (DEGs) from microarray data. The DEGs having minimum fold change (FC) of ~ 1.2 and False Discovery Rate (FDR) < 0.05 were selected for further analysis. The DEGs under comparative light conditions such as 150 vs 112, 150 vs 75, 150 vs 38, 112 vs 75, 112 vs 38 and 75 vs 38 μmol m ^−^ ^2^ s ^− ^^1^ intensities of WL were taken in consideration for the study. Largest number of DEGs were found between 150 and 38 μmol m ^−^ ^2^ s ^− ^^1^ while least number was observed in between 150 and 112 μmol m ^− ^^2^ s ^− ^^1^ light. The details of the DEGs upregulated and downregulated have been summarized in Table 1. The graphical representation of upregulated and downregulated genes for different comparative light intensities has been shown in Fig. 3.

**Table 1:** Number of Differentially Expressed Genes identified under various white light intensities

Light intensity (μmol m − ^2^ s − ^1^)	150 vs 112	150 vs 75	150 vs 38	112 vs 75	112 vs 38	75 vs 38
Total no. of genes	1014	1424	1789	1163	1518	1047
No. of genes upregulated	492	717	823	569	700	463
No. of genes downregulated	522	707	966	594	818	584

**Fig. 4:**
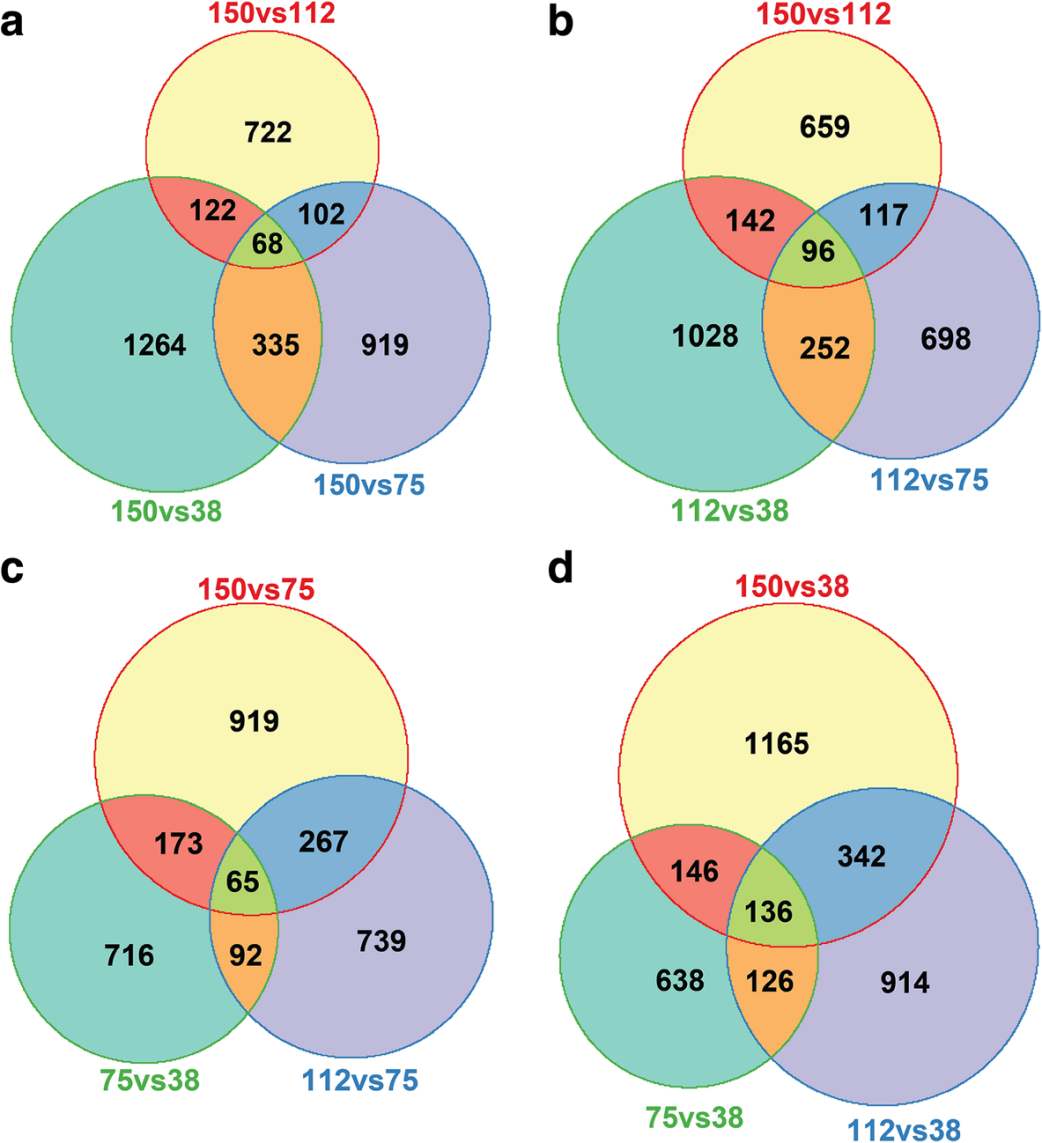
Venn diagram representation of overlapping and differential DEGs under variable white light intensity. **a** DEGs for 150 vs 112, 150 vs 75 and 150 vs 38 μmol m ^−^ ^2^ s ^−^ ^1^. **b** DEGs for 150 vs 112, 112 vs 75 and 112 vs 38 μmol m ^−^ ^2^ s ^− ^^1^. **c** DEGs for 150 vs 75, 112 vs 75 and 75 vs 38 μmol m ^− ^^2^ s ^− ^^1^. **d** DEGs for 150 vs 38, 112 vs 38 and 75 vs 38 μmol m ^−^ ^2^ s ^− ^^1^ light intensity from microarray analysis of WT root

To identify overlapping and unique genes, R program analysis was performed and presented in the Venn diagrams between 150 vs 112, 150 vs 75 and 150 vs 38 μmol m^ − ^^2^ s ^− ^^1^ light (Fig. 4a), 150 vs 112, 112 vs 75 and 112 vs 38 μmol m ^− ^^2^ s ^− ^^1^ light (Fig. 4b), 150 vs 75, 112 vs 75 and 75 vs 38 μmol m ^− ^^2^ s ^− ^^1^ light (Fig. 4c) and 150 vs 38, 112 vs 38 and 75 vs 38 μmol m ^− ^^2^ s ^− ^^1^ light (Fig. 4d) conditions. With respect to 150 μmol m ^−^ ^2^ s ^− ^^1^ intensity, under 112, 75 and 38 μmol m^ −^ ^2^ s ^− ^^1^, 68 common DEGs were identified. When light conditions of 150 vs 112, 112 vs 75 and 75 vs 38 μmol m ^− ^^2^ s ^− ^^1^ were compared, the overlapping number of DEGs was 96. When all light intensities were investigated with respect to 75 μmol m ^− ^^2^ s ^− ^^1^ light, 65 common DEGs were found. The total number of common DEGs was 136, when 150 vs 38, 112 vs 38, 75 vs 38 μmol m^ − ^^2^ s ^− ^^1^ light intensity were taken into consideration. *CYTOCHROME P450 81F2* (*CYP81F2*), At2g39445, At5g22555 and At2g44130 genes have been found to be common and altered under all six comparative light conditions. *CYP81F2* is a membrane-localized protein, known to play a role in indole glucosinolate biosynthesis and offers resistance to fungus named *Plectosphaerella cucumerina* [24]. At2g39445 encodes for phosphatidylinositol *n*-acetylglucosaminyltransferase, At5g22555 is a putative transmembrane protein while At2g44130 encodes a F-box protein *KISS ME DEADLY 3* (*KMD3*). *KMD3* has been reported to be induced by *Meloidogyne incognita* (root-knot nematode) and makes the plant susceptible towards this nematode [25]*.* The genes which were unique and common in two, three, four or five comparative light conditions have been summarized in supplementary excel files (Additional files 3 and 4).

### Gene ontology enrichment analysis of over-represented differentially expressed genes

**Fig. 5:**
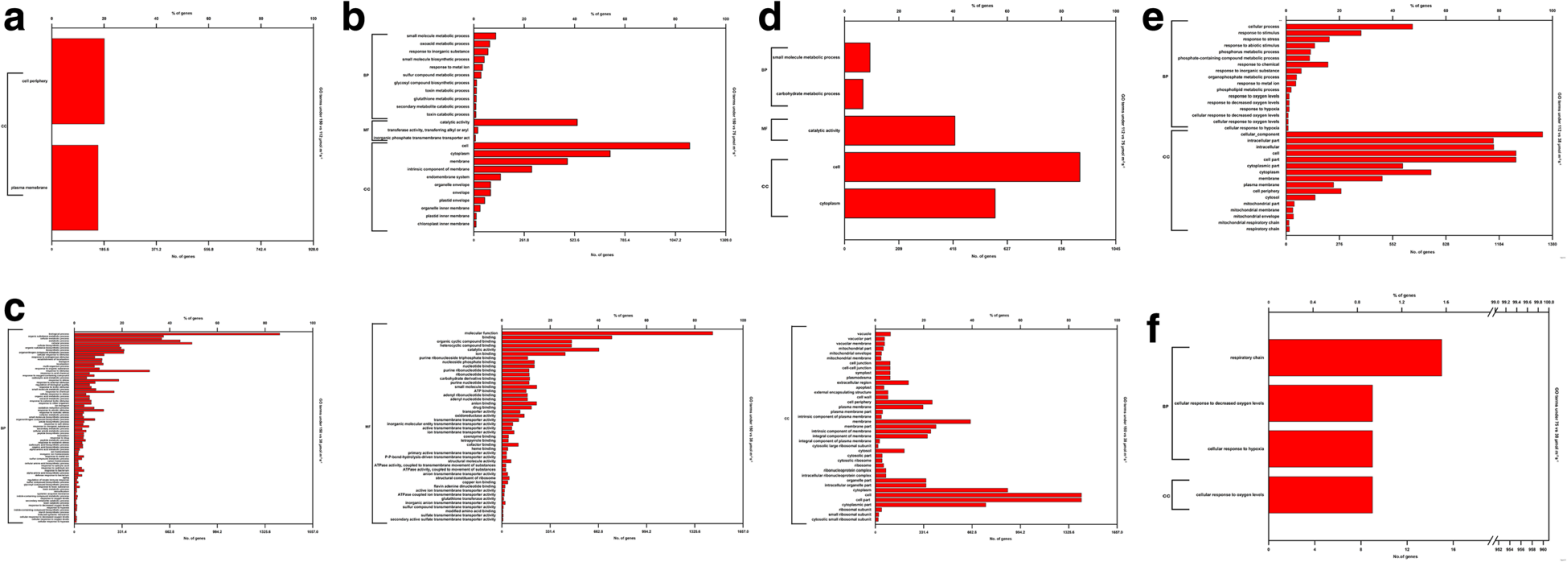
Classification of DEGs under different categories and terms of GO annotation analysis. **a** DEGs under 150 vs 112 μmol m ^− 2^ s ^− 1^ **b** DEGs under 150 vs 75 μmol m ^− 2^ s ^− 1^ **c** DEGs under 150 vs 38 μmol m ^− 2^ s ^− 1^ **d** DEGs under 112 vs 75 μmol m^ − 2^ s ^− 1^ **e** DEGs under 112 vs 38 μmol m ^− 2^ s^ − 1^ **f** DEGs under 75 vs 38 μmol m^ − 2^ s^− 1^ light intensity from microarray analysis for the functionality of DEGs in 5-days old WT root

Gene ontology (GO) enrichment analysis was performed to understand how light intensity affects different biological phenomena and processes in roots. GO analysis is based on the gene products and the related functions at molecular and cellular level with available literature databases. The analysis type used was PANTHER Over-representation Test and annotation version was GO Ontology Database [26, 27]. The categorization has been done on the basis of Fisher’s exact with FDR multiple test correction type. GO pathway analysis classifies DEGs into three categories/domains named Biological Process (BP), Molecular Function (MF) and Cellular Component (CC). The categorization is based on the gene or gene product functions and site of their functions. The BP category consists of the outcome of gene function and the major pathways involved. The MF domain represents the function of gene products at molecular level or activities of the gene at molecular level. The CC category explains about the site of gene functioning. Each category consists of various specific and broad terms based on the reported information and available database. The categorization of DEGs has been performed under 150 vs 112 μmol m^ −^ ^2^ s ^−^ ^1^ (Fig. 5a), 150 vs 75 μmol m ^− ^^2^ s ^− ^^1^ (Fig. 5b), 150 vs 38 μmol m ^− ^^2^ s ^− ^^1^ (Fig. 5c), 112 vs 75 μmol m ^− ^^2^ s ^− ^^1^ (Fig. 5d), 112 vs 38 μmol m ^− ^^2^ s ^− ^^1^ (Fig. 5e) and 75 vs 38 μmol m ^− ^^2^ s ^− ^^1^ (Fig. 5f) light intensities. The composite figure describing detailed GO analysis of DEGs has been presented in Additional file 5: Figure S3.

All the GO categories and terms which were highly enriched or over-represented under different light conditions have been summarized in Table 2. In case of 150 vs 112 μmol m ^− ^^2^ s ^− ^^1^ light condition, significant DEGs were found only under CC category, plasma membrane and cell periphery were the most affected GO terms. When the DEGs were analysed for 150 vs 75 μmol m ^−^ ^2^ s ^−^ ^1^ light, the enriched DEGs were categorized under CC and MF domains. Under this light condition, the most enriched terms were cell, cytoplasm and membrane under CC and catalytic activity was the most enriched term under MF category. The largest number of DEGs were found under 150 vs 38 μmol m ^−^ ^2^ s^ −^ ^1^ light condition and were classified under BP, CC and MF categories. The highly enriched terms under BP category were biological process, cellular process, metabolic process, cellular metabolic process and organic substance metabolic process. In this comparative light conditions, cell, cytoplasm, membrane, integral component of membrane, intrinsic component of membrane and cell periphery were the most enriched GO terms of CC category. In MF category, highly enriched terms were ion binding, heterocyclic compound binding, organic cyclic compound binding, molecular function and catalytic activity.

**Table 2:** Summary of highly affected GO categories and terms under different intensities of white light

Light intensity (μmol m ^−^ ^2^ s ^−^ ^1^)	Category	Terms	GO Accession
150 vs 112	CC	Plasma membrane	GO:0005886
		Cell periphery	GO:0071944
150 vs 75	CC	Cell	GO:0005623
		Cytoplasm	GO:0005737
		Membrane	GO:0016020
	MF	Catalytic activity	GO:0003824
150 vs 38	BP	Cellular process	GO:0009987
		Metabolic process	GO:0008152
		Cellular metabolic process	GO:0044237
		Organic substance metabolic process	GO:0071704
		Biological process	GO:0008150
	CC	Cell	GO:0005623
		Cytoplasm	GO:0005737
		Membrane	GO:0016020
		Integral component of membrane	GO:0016021
		Intrinsic component of membrane	GO:0031224
		Cell periphery	GO:0071944
	MF	Ion binding	GO:0043167
		Heterocyclic compound binding	GO:1901363
		Organic cyclic compound binding	GO:0097159
		Molecular function	GO:0003674
		Catalytic activity	GO:0003824
112 vs 75	CC	Cytoplasm	GO:0005737
		Cell	GO:0005623
	MF	Catalytic activity	GO:0003824
112 vs 38	BP	Cellular process	GO:0009987
	CC	Membrane	GO:0016020
		Cytoplasm	GO:0005737
		Cell	GO:0005623
		Intracellular	GO:0005622

The DEGs under 112 vs 75 μmol m ^−^ ^2^ s ^− ^^1^ condition were significantly categorized under BP, CC and MF, however the enriched terms were found under CC and MF categories only. Cell and cytoplasm terms were highly enriched under CC category and catalytic activity was the most affected term in MF category. In case of 112 vs 38 μmol m ^− ^^2^ s ^−^ ^1^ light, the DEGs were grouped in BP and CC categories. In BP category, the most enriched term was cellular process and under CC category, cell, cytoplasm, membrane and intracellular were the most enriched terms. The DEGs under 75 vs 38 μmol m ^−^ ^2^ s ^− ^^1^ light condition were classified under CC and BP categories and in CC category respiratory chain was the only over-represented term. The DEGs under this comparative light condition were found to be less enriched.

### Kyoto encyclopedia of genes and genomes colour pathway analysis of DEGs to investigate the gene functionality

Kyoto Encyclopedia of Genes and Genomes (KEGG) analysis represents the gene function and their utilities in biological, cellular and molecular process [28]. This analysis is based on the present database consisting of various genome sequencing, bioinformatic information etc. The DEGs obtained from microarray were further subjected to KEGG colour pathway analysis. The total number of DEGs under 150 vs 112 μmol m ^−^ ^2^ s ^−^ ^1^ light condition was 1014 and only 184 DEGs were identified with KEGG analysis. Under 150 vs 75 μmol m ^−^ ^2^ s ^−^ ^1^ light, 1424 DEGs were obtained from microarray data. In this light condition, the number of DEGs identified through KEGG colour pathway was 290. In case of 150 vs 38 μmol m ^−^ ^2^ s ^− ^^1^, 420 genes were identified by KEGG out of 1789 DEGs obtained from microarray analysis. Under 112 vs 75 μmol m ^− ^^2^ s ^−^ ^1^ light, 1163 DEGs were found from microarray, out of these, 249 genes were identified through KEGG analysis. In case of 112 vs 38 μmol m ^− ^^2^ s ^− ^^1^ light, out of 1518 DEGs only 314 genes were identified by KEGG analysis. Under 75 vs 38 μmol m ^− ^^2^ s ^− ^^1^ light, 1047 DEGs were detected from microarray and KEGG analysis identified only 172 genes.

The detailed KEGG analysis of DEGs has been presented in Table 3. This analysis showed that the metabolic pathway was the major ones affected with largest number of genes under all comparative light intensities.

**Table 3:** Categorization of DEGs on the basis of KEGG analysis

Light condition (75 μmol m ^−^ ^2^ s ^−^ ^1^)	Total no. of genes	No. of genes identified by KEGG	Major pathways involved	No. of genes in each pathway
150 vs 112	1014	184	Metabolic pathway	71
			Secondary metabolite biosynthesis	40
			Plant hormone signal transduction	19
			Protein processing in ER	15
			MAPK signaling	11
			Carbon metabolism	11
150 vs 75	1424	290	Metabolic pathway	143
			Secondary metabolite synthesis	82
			Carbon metabolism	24
			Amino acid biosynthesis	19
			Glycolysis	15
			Plant-pathogen interaction	13
			Glutathione metabolism	13
			RNA transport	13
			Starch and sucrose metabolism	13
			Oxidative phosphorylation	12
			Purine metabolism	12
			Amino sugar and nucleotide sugar metabolism	10
			Plant hormone signal transduction	10
150 vs 38	1789	420	Metabolic pathway	79
			Secondary metabolite biosynthesis	114
			Ribosome	51
			Carbon metabolism	27
			Amino acid biosynthesis	25
			Oxidative phosphorylation	21
			Phenylpropanoid biosynthesis pathway	20
			RNA transport	17
			Starch and sucrose metabolism	17
			Spliceosome	17
			Plant-pathogen interaction	17
			Glutathione metabolism	16
			Plant hormone signal transduction	15
			Endocytosis	13
			Glycine, serine and threonine metabolism	13
			Glycolysis	13
			Purine metabolism	12
			Amino sugar and nucleotide sugar metabolism	12
			Cysteine and methionine metabolism	11
			2-oxocarboxylic acid metabolism	10
			Protein processing in ER	10
			Ubiquitin mediated proteolysis	10
			Photosynthesis	10
112 vs 75	1163	249	Metabolic pathway	122
			Biosynthesis of secondary metabolites	63
			Carbon metabolism	18
			Starch and sucrose metabolism	15
			Plant hormone signal transduction	14
			Amino acid biosynthesis	12
			Protein processing in the ER	11
			Phenylpropanoid biosynthesis pathway	10
112 vs 38	1518	314	Metabolic pathway	131
			Secondary metabolite biosynthesis	59
			Ribosome	30
			Plant hormone signal transduction	27
			Carbon metabolism	22
			Oxidative phosphorylation	18
			Protein processing in ER	13
			MAPK signaling	13
			Spliceosome	12
			Purine metabolism	12
			Glyoxylate and dicarboxylate metabolism	11
			Amino acid biosynthesis pathway	10
75 vs 38	1047	172	Metabolic pathway	64
			Biosynthesis of secondary metabolite	37
			Plant hormone signaling	10
			Plant-pathogen interaction	10

### Validation of microarray result by qRT-PCR for DEGs involved in hormonal, light signaling and clock regulated pathways

We further validated the microarray result by qRT-PCR of few selected genes. Selection of genes for qRT-PCR validation was based on their i) expression level data obtained from microarray analysis and ii) significance and potential role in root development as per the KEGG pathway analysis. Therefore, the genes invoved in hormone signaling, light signaling and clock-regulated pathways were chosen for qRT-PCR validation. The root development is affected by various factors such as light, phytohormones, circadian clock etc. Photoreceptors such as PHYA and PHYB play major role in root positive phototropism [7]. PHYs have also been shown to be involved in root growth as well as its gravitropic response [5]. The phytohormones also regulate the lateral root initiation in a dose dependent manner [29]. They have also been shown to control root hair initiation and its growth [30]. High intensity of light and exogenous auxin stimulate adventitious rooting in *Eucalyptus* [31]. On the other hand, circadian clock genes have also been shown to regulate lateral root emergence [32].

**Fig. 6:**
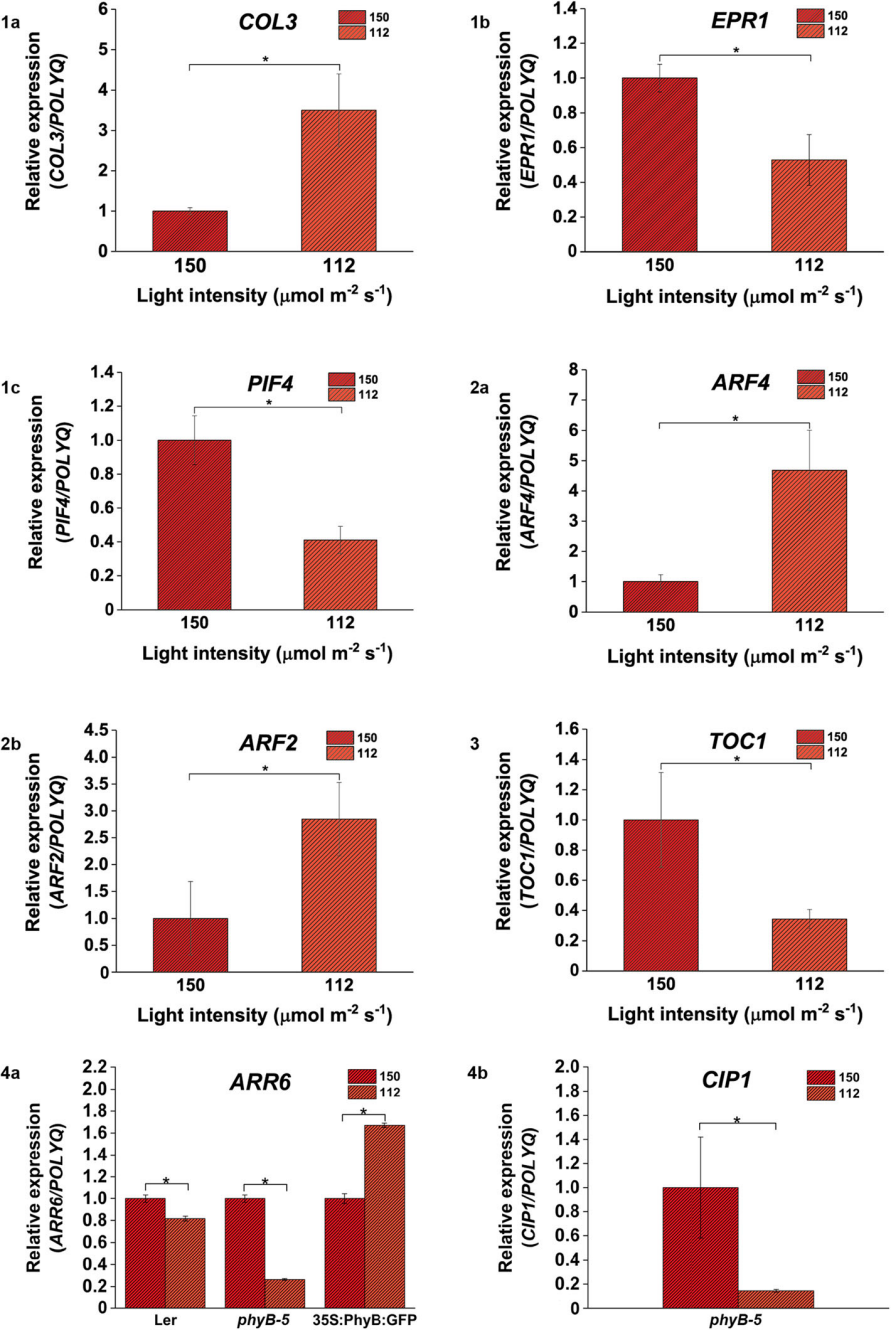
Genes involved in light, hormone and clock-regulated pathways are differentially expressed under 150 vs 112 μmol m ^− ^^2^ s ^−^ ^1^ light intensity. Light signaling genes (**1a**) *COL3*, (**1b**) *EPR1* and (6.2c) *PIF4* showed differential regulation; (**2a**) *ARF4* and (**2b**) *ARF2* genes involved in auxin signaling were differentially expressed; (**3**) *TOC1* gene showed variability in its expression in case of WT; (**4a**) *ARR6* and (**4b**) *CIP1* genes were differentially expressed in Ler, *35S::PhyBGFP*, *phyB-5* and *phyB-5* respectively under 150 vs 112 μmol m ^−^ ^2^ s ^−^ ^1^ light intensity. qRT-PCR was performed with root samples of 5-days old seedlings. Analysis has been described in the method section

**Fig. 7:**
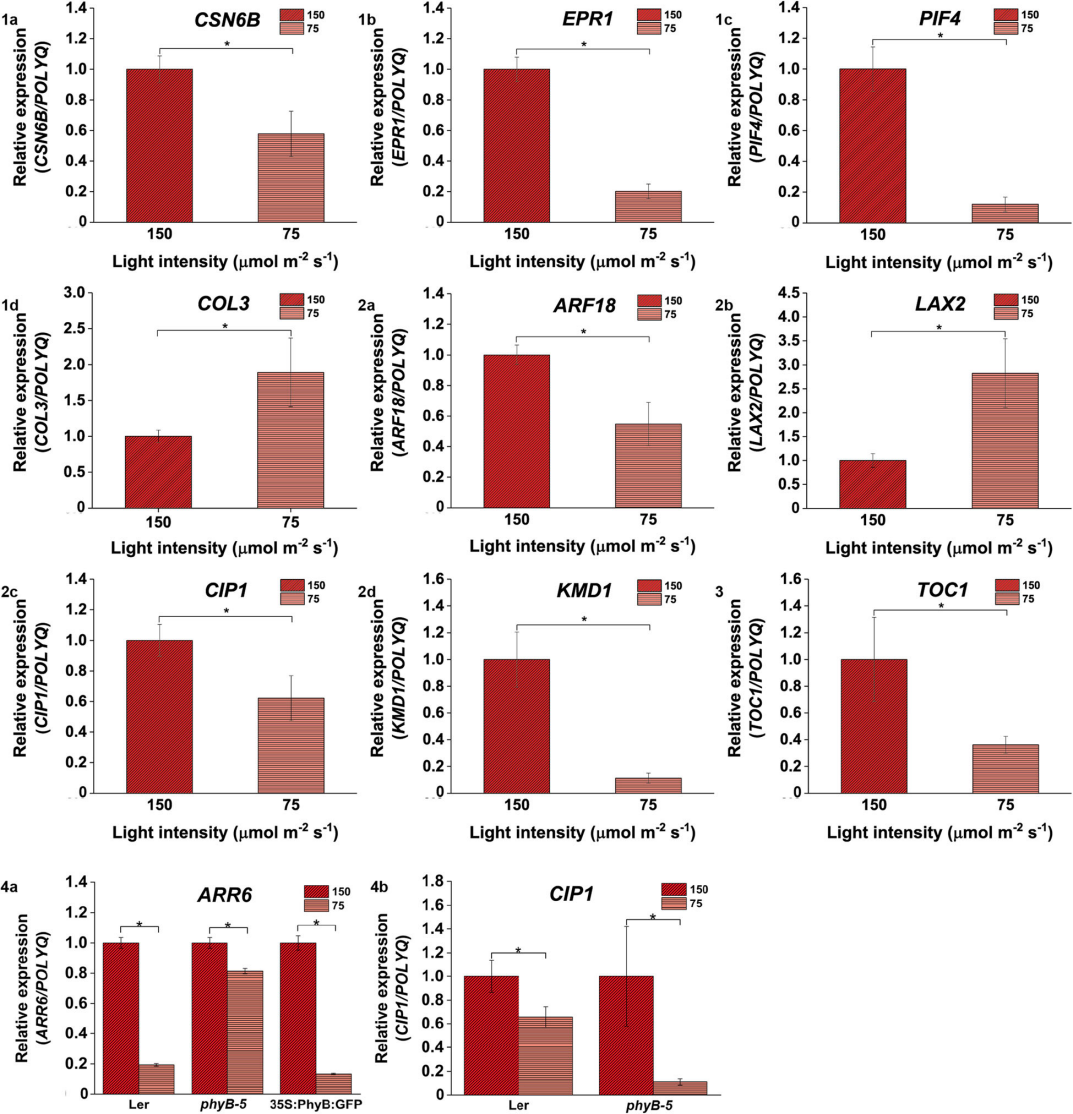
Relative expression of genes involved in light, hormone and clock-regulated pathways is influenced under 150 vs 75 μmol m ^− ^^2^ s ^−^ ^1^ light intensity. Differential expression of (**1a**) *CSN6B*, (**1b**) *EPR1*, (**1c**) *PIF4* and (**1d**)*COL3* genes; Variation in gene expression of (**2a**) *ARF18*, (**2b**) *LAX2*, (**2c**) *CIP1* and (**2d**) *KMD1* ; (**3**) *TOC1* gene regulation in WT; (**4a**) *ARR6* showed variation in its regulation in all genotypes but (**4b**) *CIP1* expression varied only in case of Ler and *phyB-5* under 150 vs 75 μmol m ^− ^^2^ s ^−^ ^1^ light intensity. qRT-PCR was performed with root samples of 5-days old seedlings. Analysis has been described in the method section

**Fig. 8:**
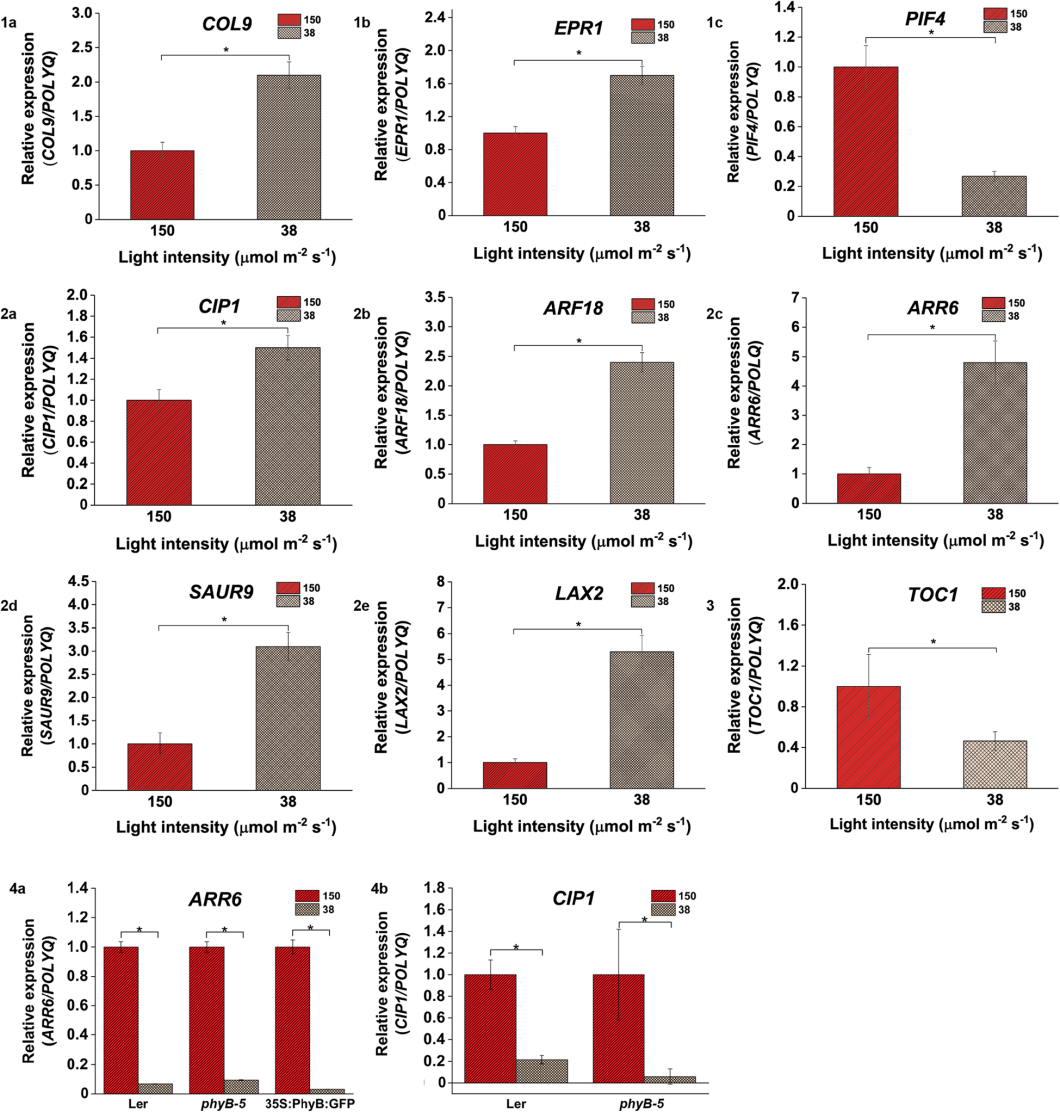
Light intensity of 150 vs 38 μmol m ^− ^^2^ s ^−^ ^1^ affects the expression profile of genes involved in light, hormone and clock-regulated pathways. Variation in expression of (**1a**) *COL9*, (**1b**) *EPR1* and (**1c**) *PIF4* candidate genes; (**2a**) *CIP1*, (**2b**) *ARF18*, (8.2c) *ARR6*, (**2d**) *SAUR9* and (**2e**) *LAX2* genes showed variable expression; (**3**) Regulation in *TOC1* gene expression in WT root; (**4a**) *ARR6* expression varied in all genotypes but (**4b**) *CIP1* expression varied only in case of Ler and *phyB-5* under 150 vs 38 μmol m ^− ^^2^ s ^−^ ^1^ light intensity. qRT-PCR was performed with root samples of 5-days old seedlings. Analysis has been described in the method section

**Fig. 9:**
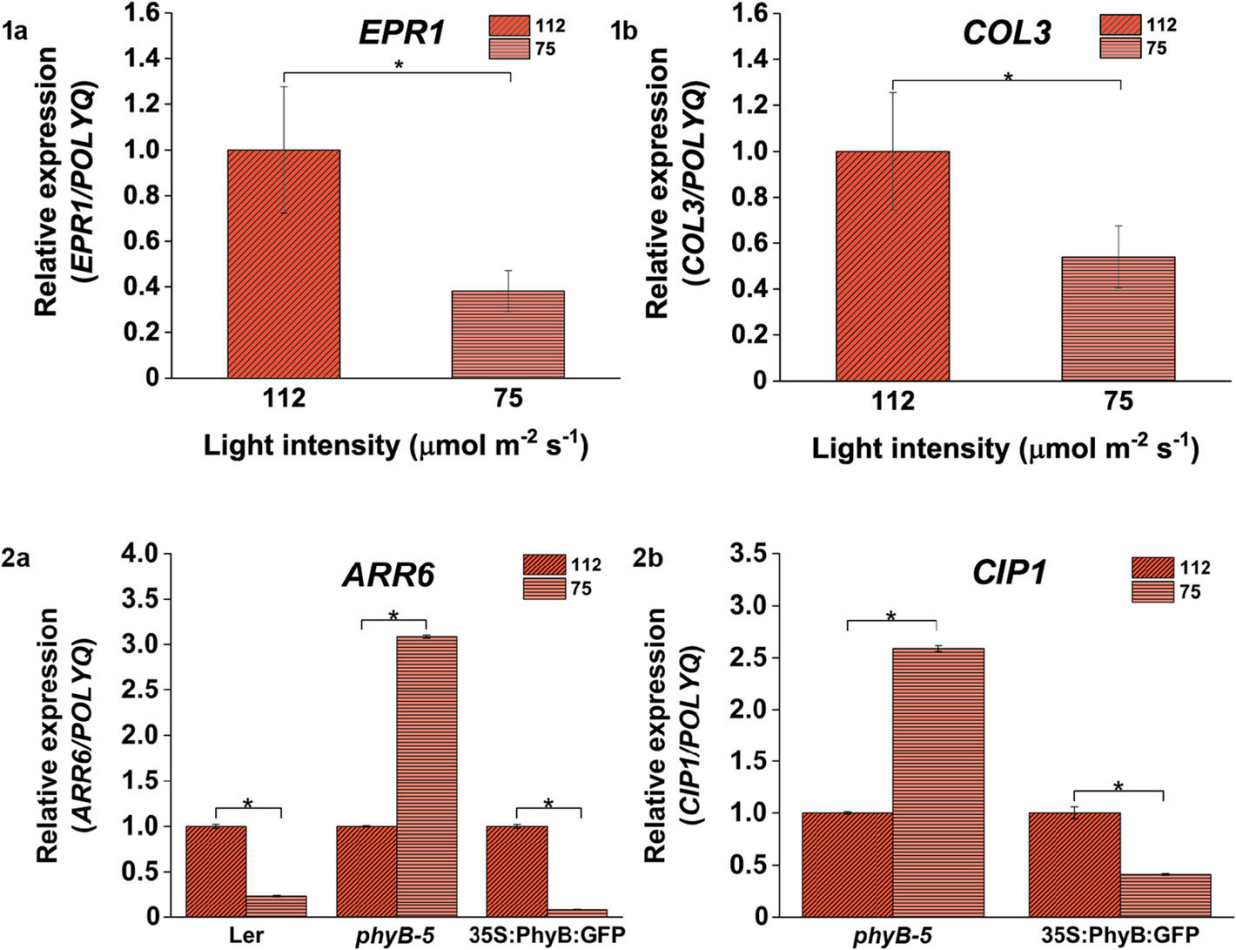
Relative expression of genes involved in light signaling pathways under 112 vs 75 μmol m ^−^ ^2^ s ^− ^^1^ light. Gene expression profiling of (**1a**) *EPR1* and (**1b**) *COL3* varied in WT root; Expression of (**2a**) *ARR6* varied in all seed lines and (**2b**) *CIP1* was differentially expressed in *35S::PhyBGFP* and *phyB-5* only under 112 vs 75 μmol m ^−^ ^2^ s ^− ^^1^ light intensity. The qRT-PCR was performed with root samples of 5-days old seedlings. Analysis has been described in the method section

**Fig. 10:**
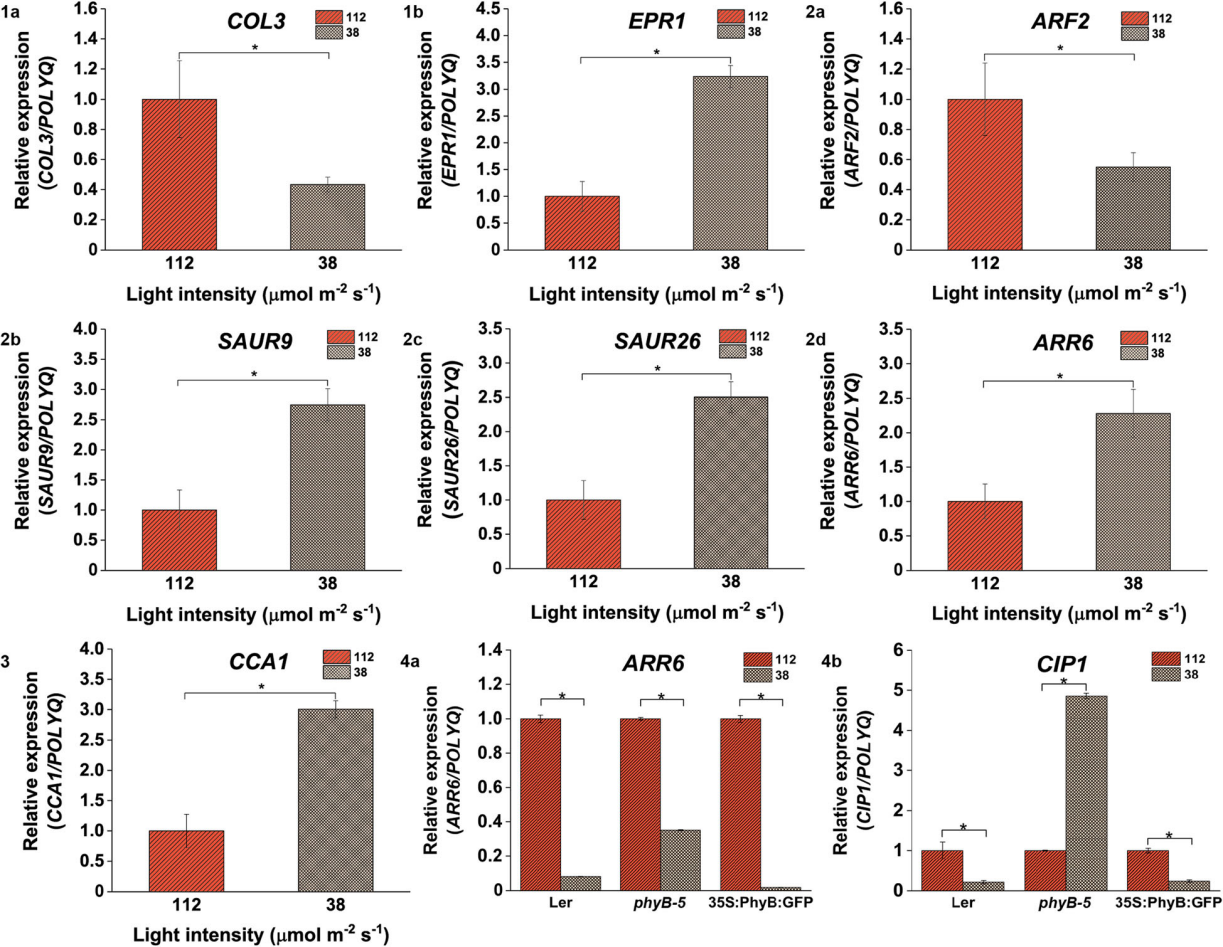
Expression profiling of genes involved in light, hormone and clock-regulated pathways is influenced under 112 vs 38 μmol m ^−^ ^2^ s ^−^ ^1^ light intensity. (**1a**) *COL3* and (**1b**) *EPR1* genes were differentially expressed; Variation in expression profile of (**2a**) *ARF2*, (**2b**) *SAUR9*, (**2c**) *SAUR26* and (**2d**) *ARR6* genes; (**3**) *CCA1* gene expression varied in WT; Expression profiling of (**4a**) *ARR6* and (**4b**) *CIP1* genes varied in all seed lines under 112 vs 38 μmol m ^− ^^2^ s ^− ^^1^ light intensity. qRT-PCR was performed with root samples of 5-days old seedlings. Analysis has been described in the method section

**Fig. 11:**
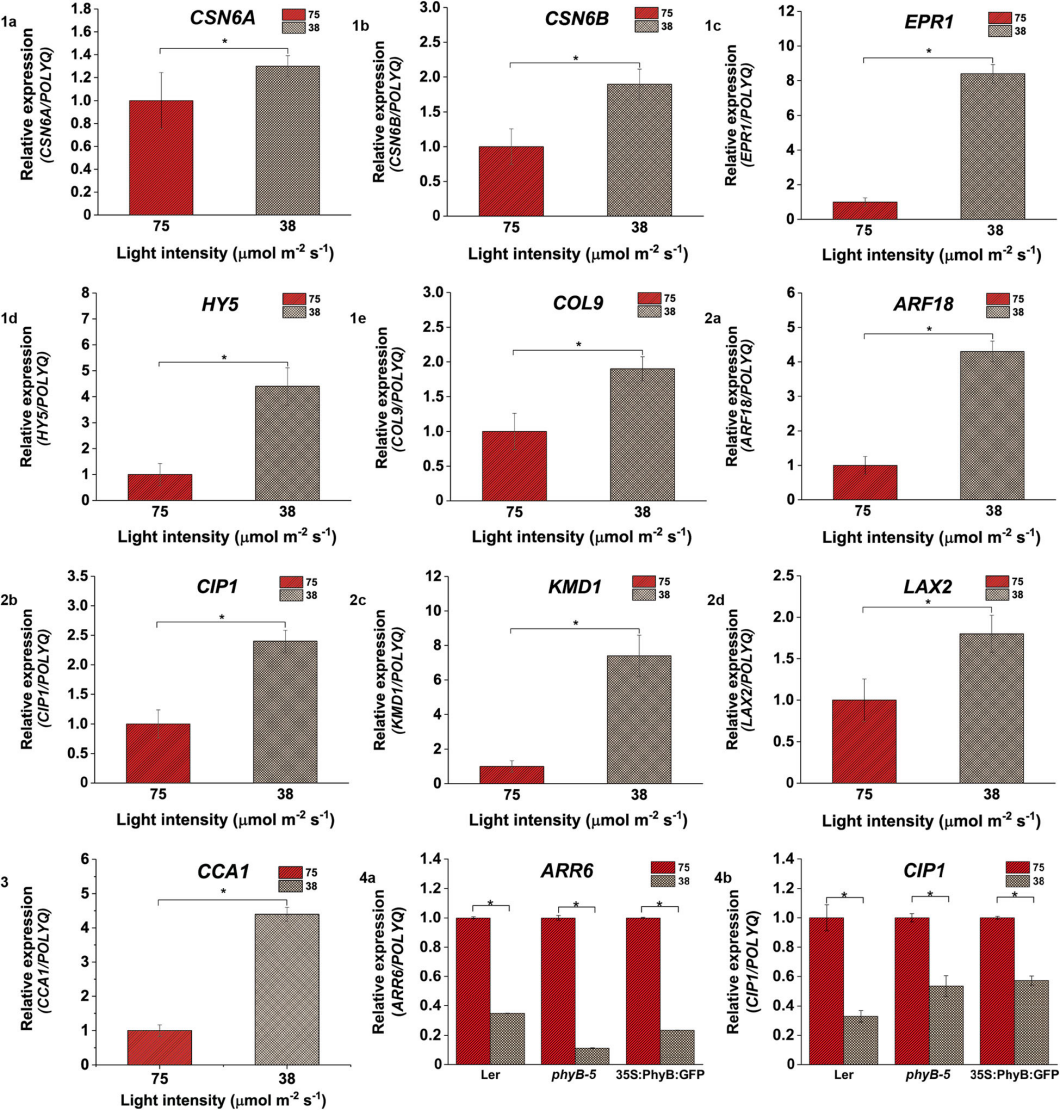
Change in relative expression of genes involved in light, hormone and clock-regulated pathways under 75 vs 38 μmol m ^−^ ^2^ s ^−^ ^1^ light intensity. Gene expression profile of (**1a**) *CSN6A*, (**1b**) *CSN6B*, (**1c**) *EPR1*, (**1d**) *HY5* and (**1e**) *COL9*  varied; (**2a**) *ARF18*, (**2b**) *CIP1*, (**2c**) *KMD1* and (**2d**) *LAX2* genes showed variable expression; (**3**) *CCA1* gene showed variable expression; Expression profiling of (**4a**) *ARR6* and (**4b**) *CIP1* genes differ in all seed lines under 75 vs 38 μmol m ^−^ ^2^ s ^− ^^1^ light intensity. qRT-PCR was performed with root samples of 5-days old seedlings. Analysis has been described in the method section

Genes such as *AUXIN RESPONSE FACTOR 2 (ARF2)*, *AUXIN RESPONSE FACTOR 4* (*ARF4)* and *AUXIN RESPONSE FACTOR 18 (ARF18)*, *LIKE AUX 2* (*LAX2)*, *SMALL AUXIN UPREGULATED RNA 9* (*SAUR9)*, *SMALL AUXIN UPREGULATED RNA 26 (SAUR26) and IAA7* were selected to evaluate the effect of WL intensity on auxin homeostasis, while, *TYPE A RESPONSE REGULATOR 6 (ARR6), KISS ME DEADLY 1(KMD1)* and *COP1 INTERACTING PROTEIN 1* (*CIP1)* were analysed to understand the impact of WL intensity on cytokinin and ABA signaling, respectively. Genes involved in light signaling such as *PHYTOCHROME RAPIDLY REGULATED 2* (*PAR2)*, *HY5*, *PHYTOCHROME INTERACTING FACTOR 4* (*PIF4)*, *EARLY PHYTOCHROME RESPONSE 1* (*EPR1)*, *CONSTANS LIKE 3* (*COL3)*, *CONSTANS LIKE 9* (*COL9)*, *COP9 SIGNALOSOME COMPLEX SUBUNIT 6A* (*CSN6A)* and *COP9 SIGNALOSOME COMPLEX SUBUNIT 6B* (*CSN6B)* were selected to investigate the correlation of WL intensities with expression level of downstream light signaling components. Three clock-associated genes such as *CIRCADIAN CLOCK-ASSOCIATED 1* (*CCA1*), *TIMING OF CAB EXPRESSION 1* (*TOC1*) and *PSEUDO RESPONSE REGULATOR 9* (*PRR9*) were also chosen to understand the response of light quantity on circadian clock regulation. The possible functions of all the above-mentioned genes and respective primers have been compiled in Table 4 and supplementary file (Additional file 6) respectively. The qRT-PCR was performed with the same RNA samples used for microarray analysis of WT. The gene expression of all the light signaling, hormone signaling and clock-regulated genes mentioned earlier were analysed for 150 vs 112, 150 vs 75, 150 vs 38, 112 vs 75, 112 vs 38 and 75 vs 38 μmol m^ −^ ^2^ s ^−^ ^1^ comparative light intensity (Figs. 6, 7, 8, 9, 10 and 11). The FC of gene expression data has been summarized in Table 5.

**Table 4:** List of gene functions

Gene names	Gene function
PAR2	Negative regulator of shade avoidance syndrome responses and transcriptional repressor of SAUR15 and SAUR68 [33].
ARR6	Type-A response regulator, acts as negative regulator of cytokinin signaling [34].
HY5	Transcription factor playing downstream to photoreceptor signaling, promotes photomorphogenesis. It is also involved in root greening and gravitropism [15, 35].
ARF18	Auxin response factor acting as transcriptional repressor for auxin responsive genes [36].
ARF2	Auxin response factor acting as transcriptional repressor for auxin responsive genes [37].
ARF4	Auxin response factor acting as transcriptional repressor for auxin responsive genes [38].
KMD1	Type-A response regulator which targets type-B response regulator and acts as negative regulator of cytokinin signaling [39].
PIF4	Transcription factor playing negatively in phytochrome signaling pathway [40].
IAA7	Auxin inducible gene which negatively regulates auxin signaling [41].
EPR1	Transcriptional factor playing role in *PHYA*-mediated cotyledon opening and it is regulated by circadian clock [42].
COL3	Zn finger protein which is a positive regulator of red light signaling and photomorphogenesis. Also regulates root and shoot development [43].
SAUR9	Early auxin responsive gene induced on auxin application [44].
SAUR26	Early auxin responsive gene induced on auxin application [45].
LAX2	Auxin influx carrier involved in leaf venation [46].
COL9	CONSTANS like protein, acts as negative regulator of flowering [46].
CIP1	COP1 interaction protein, plays positive role in ABA response [47].
CSN6A	Component of COP 9 signalosome complex regulating ubiquitin conjugation pathway and plays role in repression of photomorphogenesis in dark [48].
CSN6B	Component of COP 9 signalosome complex regulating ubiquitin conjugation pathway and plays role in repression of photomorphogenesis in dark [48].
CCA1	Morning loop gene which functions along with LHY1 and it represses TOC1 [49].
TOC1	Evening complex gene, regulates plant fitness by controlling the clock output through repressing morning and evening loop of circadian clock [50].
PRR9	Temperature sensitive component of circadian clock, interacting with TOC1 [51].

**Table 5:** Relative expression of selected genes analysed by qRT-PCR

Gene names	Light Intensity (μmol m ^−^ ^2^ s ^−^ ^1^)	Fold change
ARR6	150 vs 38	4.7
	112 vs 38	2.2
HY5	75 vs 38	4.4
ARF18	150 vs 75	−1.8
	150 vs 38	2.4
	75 vs 38	4.3
ARF2	150 vs 112	2.8
	112 vs 38	− 1.8
ARF4	150 vs 112	4.6
KMD1	150 vs 75	−8.7
	75 vs 38	7.4
PIF4	150 vs 112	−2.4
	150 vs 75	−8.3
	150 vs 38	−3.7
EPR1	150 vs 112	−1.8
	150 vs 75	−4.9
	150 vs 38	1.7
	112 vs 75	− 2.6
	112 vs 38	3.2
	75 vs 38	8.4
COL3	150 vs 112	3.5
	150 vs 75	1.8
	112 vs 75	−1.8
	112 vs 38	− 2.3
SAUR9	150 vs 38	3.1
	112 vs 38	2.7
SAUR26	112 vs 38	2.5
LAX2	150 vs 75	2.8
	150 vs 38	5.3
	75 vs 38	1.8
COL9	150 vs 38	2.1
	75 vs 38	1.9
CIP1	150 vs 75	−1.6
	150 vs 38	1.5
	75 vs 38	2.4
CSN6A	75 vs 38	1.3
CSN6B	150 vs 75	−1.7
	75 vs 38	1.9
CCA1	112 vs 38	3
	75 vs 38	4.3
TOC1	150 vs 112	−2.9
	150 vs 75	−2.7
	150 vs 38	−2.1

qRT-PCR analysis showed that in comparison to 150 μmol m ^−^ ^2^ s ^−^ ^1^ light intensity, under 112 μmol m ^− ^^2^ s ^−^ ^1^, a positive regulator of red light signaling, *COL3* was significantly upregulated (Fig. 6.1a) whereas other transcription factors involved in PHY signaling such as *EPR1* and *PIF4* were downregulated (Fig. 6.1b and .1c). Auxin responsive factors such as *ARF2* and *ARF4* were upregulated under the mentioned light intensity (Fig. 6.2a and .2b). The clock-regulated genes such as *TOC1* was downregulated under 112 μmol m ^− ^^2^ s ^− ^^1^ light intensity compared to 150 μmol m ^−^ ^2^ s ^−^ ^1^ light intensity (Fig. 6.3). Under 75 μmol m ^− ^^2^ s^ −^ ^1^ compared to 150 μmol m ^−^ ^2^ s ^−^ ^1^ intensity of light, the expression level of light signaling genes such as *CSN6B*, *EPR1*, and *PIF4* genes was reduced (Fig. 7.1a, .1b and .1c) however, *COL3* expression was enhanced (Fig. 7.1d). Auxin influx carrier, *LAX2* was upregulated (Fig. 7.2b) whereas *ARF18*, *CIP1* and *KMD1* were downregulated (Fig. 7.2a, .2c and .2d) under this comparative light condition. *TOC1* was downregulated under 75 μmol m ^−^ ^2^ s ^−^ ^1^ in comparison to 150 μmol m ^−^ ^2^ s ^−^ ^1^ intensity (Fig. 7.3). Under 38 μmol m ^−^ ^2^ s ^−^ ^1^ light in comparison to 150 μmol m ^− ^^2^ s ^−^ ^1^, the expression of light signaling genes like *COL9* and *EPR1* was upregulated (Fig. 8.1a and .1b) whereas *PIF4* expression was downregulated (Fig. 8.1c). On the other hand, genes involved in hormone signaling such as *CIP1*, *ARF18*, *ARR6, SAUR9,* and *LAX2* were upregulated (Fig. 8.2a, .2b, .2c, .2d and .2e). *TOC1* was downregulated under 38 μmol m ^−^ ^2^ s ^−^ ^1^ light as compared to 150 μmol m ^−^ ^2^ s ^−^ ^1^ (Fig. 8.3). Overall, it was observed that *TOC1* is downregulated in all lower light intensities compared to highest intensities.

In comparison to 112 μmol m ^−^ ^2^ s ^−^ ^1^, under 75 μmol m ^−^ ^2^ s^ −^ ^1^ light condition, the expression of *EPR1* and *COL3* was downregulated (Fig. 9a and b). There was no significant change observed in the expression pattern of hormonal signaling and clock-regulated genes under this comparative light condition. With respect to 112 μmol m ^−^ ^2^ s ^−^ ^1^ under 38 μmol m ^− ^^2^ s ^−^ ^1^ light intensity, *COL3* expression was reduced whereas *EPR1* gene was enhanced (Fig. 10.1a and .1b). When hormone signaling pathway genes were focused, it was observed that the expression of *SAUR9*, *SAUR26* and *ARR6* was upregulated (Fig. 10.2b, .2c and .2d) whereas *ARF2* expression was downregulated (Fig. 10.2a). The expression of clock regulated gene such as *CCA1* was upregulated under this comparative light condition (Fig. 10.3).

In comparison to 75 μmol m^ −^ ^2^ s^ −^ ^1^ under low light intensity of 38 μmol m ^− ^^2^ s ^−^ ^1^, the expression of *CSN6A*, *CSN6B*, *EPR1, HY5* and *COL9* genes was higher (Fig. 11.1a, .1b, .1c, .1d and .1e) and hormone pathway genes such as *ARF18*, *CIP1*, *KMD1* and *LAX2* were also upregulated (Fig. 11.2a, .2b, .2c and .2d). *CCA1* expression was also shown to be upregulated under this low light condition (Fig. 11.3). The genes which were induced or repressed under low light intensity in comparison to high intensity of light have been summarized in Table 6.

**Table 6:** Genes induced and repressed in roots under lower light versus higher light intensity

Light intensities (μmol m ^− ^^2^ s ^−^ ^1^)	Genes Upregulated	Genes downregulated
150 vs 38	*COL9*, *EPR1*, *CIP1*, *ARF18*, *ARR6*, *SAUR9* and *LAX2*	*PIF4* and *TOC1*
112 vs 38	*EPR1*, *SAUR9*, *SAUR26*, *ARR6* and *CCA1*	*COL3* and *ARF2*
75 vs 38	*CSN6A*, *CSN6B*, *EPR1*, *HY5*, *COL9*, *ARF18*, *CIP1*, *KMD1*, *LAX2* and *CCA1*	

Further to validate the transcriptomics data, we performed the qRT-PCR for *ARR6* and *CIP1* genes in *Landsberg erecta* (Ler), constitutively overexpressor (*35S::PhyB:GFP*) and mutant (*phyB-5*) lines of PHYB in Ler background. qRT-PCR was performed with root samples of 5-days old seedlings grown under all four different light intensities. It has been observed that the expression of *ARR6* and *CIP1* was downregulated in case of Ler and *phyB-5* under 112 μmol m ^−^ ^2^ s ^−^ ^1^ in comparison to 150 μmol m ^−^ ^2^ s ^−^ ^1^ light (Fig. 6.4a and .4b) whereas *ARR6* has been shown to be significantly upregulated in case of *35S::PhyBGFP* (Fig. 6.4b). When the expression of these two genes were analysed in presence of 75 μmol m ^−^ ^2^ s ^−^ ^1^ in comparison to 150 μmol m ^−^ ^2^ s^−1^ light intensity, *ARR6* was downregulated in all three genotypes (Fig. 7.4a) and *CIP1* showed downregulation only in case of *phyB-5* genotype (Fig. 7.4b)*.* Similarly, under low light of 38 μmol m ^−^ ^2^ s ^−^ ^1^ intensity in comparison to 150 μmol m^ −^ ^2^ s ^− ^^1^, both these genes were downregulated. However, *CIP1* did show a significant downregulation only in case of Ler and *phyB-5* (Fig. 8.4a and .4b).

When the relative expression pattern of *ARR6* and *CIP1* was analysed under 75 μmol m ^− ^^2^ s ^−^ ^1^ in comparison to 112 μmol m ^−^ ^2^ s^−1^light intensity, *ARR6* was found to be downregulated in Ler and *35S::PhyBGFP* whereas it was upregulated in case of *phyB-5* (Fig. 9.2a)*.* On the other hand, *CIP1* was upregulated in *phyB-5* but downregulated in case of *35S::PhyBGFP* (Fig. 9.2b)*.* Under 38 μmol m^ − ^^2^ s ^− 1^ as compared to 112 μmol m ^−^ ^2^ s^−1^ light intensity, *ARR6* expression was downregulated in all genotypes tested (Fig. 10.4a). *CIP1* was downregulated in case of Ler and *35S::PhyBGFP* whereas upregulated in *phyB-5* (Fig. 10.4b)*.* Both the genes were downregulated under 38 μmol m ^−^ ^2^ s ^− ^^1^ in comparison to 75 μmol m ^−^ ^2^ s ^−^ ^1^ light intensity in all three genotypes (Fig. 11.4a and .4b). These data suggested that *ARR6* and *CIP1* are differentially regulated by PHYB under variable WL intensities.

## Discussion

Although earlier studies have investigated the effect of light intensity as well as light quality on root development, yet the molecular players that fine tune the output response are poorly understood [1, 17]. Light signaling mediated through phytohormones modulate plant development [52]. Under shade, where the R:FR ratio is very low, the light is primarily sensed by PHYs and their downstream factors [53]. This leads to change in plant phenotype such as elongated hypocotyl, longer petiole, small leaves, apical hook formation, etc. On the other hand, phytohormones such as gibberellin (GA), auxin (IAA), ethylene (ET) and brassinosteroid (BR) have been shown to regulate plant development under relative dark and shade conditions. GA promotes hypocotyl elongation and suppresses other photomorphogenic features, these phenotypic differences appear through inactivation of *HY5* and other light signaling transcription factors such as *PIFs*. Light controls GA synthesis by downregulating GA biosynthetic enzymes and stimulating GA-inactivation enzymes [54]. It has also been reported that shade (low R:FR) induces hypocotyl growth via enhanced *TRYPTOPHAN AMINOTRANSFERASE OF ARABIDOPSIS 1* (*TAA1*)-dependent auxin biosynthesis [53]. *PIF5* promotes ethylene-mediated apical hook formation, one of the characteristics of shade avoidance syndrome in dark [55]. Along with light, circadian clock also regulates phytohormone signaling and synthesis. Ethylene production is regulated by circadian clock genes such as *TOC1* and *CCA1*, similarly *ACC SYNTHASE 8* (*ACS8*) is also controlled by light and circadian clock [56]. Auxin signaling and responses are also regulated by circadian clock [57]. It has been reported that cytokinin t-zeatin treatment induces expression of *TOC1*, *GIGANTEA* (*GI*) in morning and *CCA1* in the evening. Active NAA or ABA treatment has been shown to downregulate *CCA1* expression in morning [58]. *TOC1* and other circadian clock-associated genes have been reported to control the expression of genes involved in GA biosynthesis [59]. Clock regulates the transcript level of *PIF4* and *PIF5* genes [60]. In the present work, we have shown that different WL intensities (150, 112, 75 and 38 μmol m ^−^ ^2^ s ^−^ ^1^) show variable effects on the root development in case of WT and PHY mutants. Light quantity influenced the root architecture as well as the expression profile of the genes playing role in light signaling, phytohormone related and clock-regulated pathways. It has been reported that, although only aerial shoot portion perceive light, some amount of light gets translocated down through light piping and influences root patterning [9]. In the current work, we explored the candidate genes involved in light intensity-based root patterning over direct exposure of seedlings to light.

### Primary, lateral and adventitious roots are affected by different intensities of white light

Different intensities of light as well as their quality have been shown to affect root development [20–22, 17]. PHYs sense the quality as well quantity of light and have been shown to influence phototropism, gravitropism and elongation of root [5]. Under shade condition, where the ratio of R:FR is reduced, the hypocotyl elongates, however, the effect of shade on root development is poorly understood [61]. We have shown here that under different light intensity, the root patterning changes significantly. Primary root elongation was more influenced in case of PHY mutants*.* Higher light intensity (150 μmol m ^−^ ^2^ s ^−^ ^1^) induced larger number of lateral as well as adventitious roots. PHYB predominantly affected the adventitious root growth whereas PHYA has been shown to be involved in primary root growth based on variable light intensity. Both PHYA and PHYB are involved in sensing different intensities of WL that affect the root architecture accordingly. The analysis of root wave, root coiling, root hair density etc.*,* need to be carried out in detail in PHY mutants under different WL intensity. These aspects should also be addressed under different light intensities of monochromatic light to understand the correlation of different quality and quantity of light on root architecture.

### Light intensity influences root development and also affects the gene expression profile in root

When light signal translocates through the shoot to the root or seedling directly exposed to light, the gene expression profile of root changes differently. It has been shown that the spatial expression profile of photoreceptors changes throughout the root when shoot is directly exposed to light [23]. With microarray analysis, we found about 5243 DEGs under four different light intensities (150, 112, 75 and 38 μmol m ^−^ ^2^ s ^−^ ^1^) and highest and lowest number of DEGs were detected under 150 vs 38 μmol m ^−^ ^2^ s ^−^ ^1^ light condition and 150 vs 112 μmol m ^−^ ^2^ s^ −^ ^1^ light intensity respectively. The change of light intensity from high to low, severely affected the gene expression pattern of root whereas the slight change of 150 to 112 μmol m ^−^ ^2^ s^ −^ ^1^ light didn’t have significant effect on gene expression profile. Interestingly, *CYP81F2* (Cytochrome P450 in indole glucosinolate biosynthesis), At2g39445 (phosphatidylinositol *n*-acetylglucosaminyltransferase), At5g22555 (transmembrane protein) and At2g44130 (F-box protein) genes were altered in all comparative light intensity. The functions of these candidate genes should be studied in detail as this can correlate the light intensity-based root development with glucosinolate biosynthesis, nucleotide sugar and glycolipid signaling and SCF ubiquitin-mediated pathways.

### GO enrichment and KEGG colour pathway analysis of DEGs highlight the potential role of specific pathways involved in light intensity-based root development

GO analysis suggested about functionality of the genes altered under variable light intensities, their specific locations and their involvement in the major biological, cellular and molecular processes. The change in light intensity affected the root development possibly by influencing the catalytic activities of the genes present in root. Different intensities of light also altered the expression of gene predominantly localized in cytoplasm, cell periphery and membrane part of the cell. KEGG analysis has shown that various pathways such as metabolic, secondary metabolite biosynthesis, ribosome-mediated, carbon metabolism, plant hormone signaling, protein processing in ER, MAPK signaling, amino acid biosynthesis, starch and sucrose metabolic, oxidative phosphorylation, purine metabolic, amino and nucleotide sugar metabolic, etc. were altered in roots on irradiation of different WL intensities.

### Light intensity affects the expression pattern of genes involved in the light signaling, hormone related and clock-regulated pathways in roots

Earlier reports showed that light affects root development through photoreceptors. PHYA promotes root elongation under R, FR, B while PHYB enhances root length under R light only [61, 18]. Lateral root growth is promoted by PHYA, PHYB and PHYE whereas the high light sensor PHYD inhibits this phenomenon [4]. Root hair formation is also promoted by light where PHYA and PHYB have been shown to stimulate root hair initiation [62]. Therefore, few genes known to play major role in light signaling were analysed to investigate that how they are regulated by different light intensities. Remarkably, the expression of PHYA and PHYB was unaltered under all tested light intensities. This could be possibly because of post translational modification of PHYs under these light intensities, which may further lead to their instability and degradation in roots. It also indicates that the expression level of PHYs may change under other intensities of light not investigated in the present work. Furthermore, light dependent mRNA stability and subsequent splicing events adding to another layer of complex regulation may be possible. Then, expression profile of other light signaling genes were analyzed and it was observed that the expression of *PIF4* was maximum at higher intensity of 150 μmol m ^−^ ^2^ s ^−^ ^1^ light whereas the expression of *HY5*, *CSN6A*, *CSN6B*, *EPR1* and *COL9* was highest under 38 μmol m ^−^ ^2^ s^ −^ ^1^ light. The expression of positive regulator of red light signaling and suppressor of flowering, *COL3* was observed to be highest under 112 μmol m ^−^ ^2^ s ^−^ ^1^ light. These genes are most probable candidate for sensing the light quality along with quantity to affect the root architecture. Expression of PHYs didn’t change, however, their downstream genes were found to be altered with different light intensities irradiated on root. Light affects the hormone synthesis, signaling and transport and known to be involved in root development [63]. Hence, few genes involved in auxin, cytokinin and ABA pathways were analysed in this current work. The expression level of auxin responsive factors such as *ARF2* and *ARF4* was observed to be maximum under 112 μmol m ^−^ ^2^ s ^−^ ^1^ light. Auxin influx carrier; *LAX2*, negative regulator of cytokinin signaling; *ARR6* and a positive regulator of ABA signaling; *CIP1* have shown maximum expression under low light intensity of 38 μmol m ^−^ ^2^ s ^− ^^1^. Specific light intensity may alter certain metabolic processes that might account for the observed output gene regulation. Early auxin-responsive genes such as *SAUR9* and *SAUR26* were upregulated with decreasing light intensity and their expression was maximum at 38 μmol m ^−^ ^2^ s ^−1^ light. This differential gene expression profiling showed that various hormonal pathways are also influenced by different light quantity that further lead to the differences in root architecture. Circadian clock has a major role in plant development and it has been reported to affect root growth [64, 32]. Clock genes such as *CCA1*, *PRR9* and *TOC1* were analyzed in this current work and it was observed that, the expression of *CCA1* and *TOC1* was maximum under 38 μmol m ^−^ ^2^ s ^−^ ^1^ and 150 μmol m ^−^ ^2^ s ^−^ ^1^ light intensity respectively. They are associated with evening and morning complex and make sense to peak at low light intensities normally represented as dawn and dusk. However, their upregulation at high light intensity may be a result of light stress response. Strikingly, expression of *PRR9* was not significantly changed under any light condition, suggesting that *PRR9* oscillation is robust and not influenced by the concerned component. It shows that both light intensity and clock-regulated pathways interact to regulate the root architecture. A detailed study of these genes needs to be done for their involvement in intensity dependent root growth. Difference in light intensity alters various light signaling, hormone associated and clock-regulated genes localized in root leading to change in its phenotype. Most of the genes analysed in by qRT-PCR had shown maximum expression at 38 μmol m ^−^ ^2^ s ^−^ ^1^ light. Very few genes analysed in this study such as *PIF4* and *TOC1* showed highest expression at higher light intensity of about 150 μmol m ^−^ ^2^ s ^−^ ^1^ light. From the transcriptome validation data, it was observed that expression profile of few of the genes such as *ARR6* and *CIP1* are light intensity as well as PHYB dependent. In Ler, *ARR6* and *CIP1* showed antagonistic regulation with respect to WT under few light intensities. As under 38 μmol m ^− ^^2^ s ^−^ ^1^, *ARR6* was downregulated in case of Ler when compared with 150 and 112 μmol m ^−^ ^2^ s ^−^ ^1^ whereas it has shown upregulation in WT. Similarly, *CIP1* showed downregulation under 38 μmol m ^−^ ^2^ s ^−^ ^1^ in comparison to 150 and 75 μmol m ^−^ ^2^ s ^−^ ^1^ light in Ler on the other hand, it was upregulated in case WT. These variable gene expression in different genetic lines suggest that the light intensity mediated gene expression in roots may be partly dependent on ecotypes. Similar reports are available, which have shown that timing to flower varies in different ecotypes. Under variable light intensities, the net amount of Pr and Pfr varies and are most likely regulated in spatio-temporal manner. The amount of endogenous level of active and inactive forms of photoreceptor at a given temperature can perhaps add an extra layer of complexity to generate a transcriptomics pattern as a read-out.

The investigation of genes involved in other phytohormone signaling, synthesis and transport under variable light intensities could be carried out. Few of the light signaling genes such as *PIF5*, *FAR-RED ELONGATED HYPOCOTYL 1* (FHY1), *FHY1-LIKE* (*FHL*), *CONSTITUTIVE PHOTOMORPHOGENESIS 1* (*COP1*) etc. could also be analysed with respect to light intensity based root growth. The expression profiling of the candidate genes playing role in shade avoidance syndrome like *HY5*, *LONG HYPOCOTYL IN FAR-RED LIGHT* 1 (*HFR1*), *Arabidopsis thaliana HOMEOBOX PROTEIN 4* (*ATHB4*) etc. could also be studied under changing light intensity [53]. The expression of these genes could be analysed in the shoot as well as root under the four WL intensities mentioned or other suitable intensities. These studies could be extended by performing different sets of experiments with variable intensity of R, FR, B light and then analyzing the expression of genes involved in light, phytohormone signaling, clock regulation as well as other related pathways.

## Conclusion

Our current work showed the effect of different WL intensity on the root phenotype of WT as well as *phyA* and *phyB* mutants. The primary root length was shorter in case of *phyA-211* under all four variable light intensities, however under higher intensities of 112 and 150 μmol m ^−^ ^2^ s ^−^ ^1^ both the *PHY* mutants had shown shorter primary root in comparison to WT. *phyB-9* had shown more adventitious roots in comparison to *phyA-211* and WT under same light intensity. Under 38 and 75 μmol m ^−^ ^2^ s ^−^ ^1^ light intensity, *phyA-211* had lesser number of lateral roots whereas under 112 μmol m ^−^ ^2^ s ^−^ ^1^ light, *phyB-9* showed lesser number of lateral roots in comparison to *phyA-211* and WT. Both adventitious as well as lateral root number were similar in all genotypes under 150 μmol m ^−^ ^2^ s ^−^ ^1^. Higher intensity of WL such as 150 μmol m ^− ^^2^ s ^−^ ^1^ and lower intensity of light such as 38 μmol m ^−^ ^2^ s ^−^ ^1^ had the most significant effects on the gene expression profile of root as the number of DEGs was found to be highest in case of 150 vs 38 μmol m ^−^ ^2^ s ^−^ ^1^ light. KEGG colour analysis pathway has shown that most of the genes found from microarray belonged to metabolic, secondary metabolite synthesis, carbon metabolic and plant hormone signaling pathway. Genes such as *PIF4*, *EPR1*, *COL3*, *CIP1*, *TOC1* etc. were shown to be differentially expressed under almost all WL intensities. These genes may act as possible candidates in correlation of light intensity dependent root development. This study showed that different quantity of light affects the transcript abundance of light, hormone and clock pathway genes in the root tissues, indicating their potential role in light intensity mediated root development. Transcriptomics profiling using laser capture microdissection at single cell/tissue level could be valuable and would add in depth understanding of the regulatory network.

## Methods

### Plant material and growth conditions

Columbia Wild type (WT), phytochrome mutants such as *phyB-9* and *phyA-211* seed lines of *Arabidopsis thaliana* were used for root architectural studies. For microarray analysis, WT root samples were used. The initial qRT-PCR validation was performed with WT and for further transcriptome validation, Landsberg *erecta* (Ler) wild type, overexpressor line, *35S::PhyBGFP* (*Landsberg erecta*) and mutant line, *phyB-5* (*Landsberg erecta*) of PHYB were used. These seed lines were a kind gift from Prof. Eberhard Schäfer, Albert Ludwigs University of Freiburg, Germany [65, 66]. The seeds were surface sterilized with 70 and 100% ethanol sequentially and dried under sterile bench. The sterilized seeds were sown on half-strength Murashige and Skoog salt (Himedia, Cat. No. PT021) supplemented with 1% sucrose (w/v) (MP biomedical, Cat. No. 194018) and 0.8% agar (w/v) (Himedia, Cat. No. PCT0901) in square plates and then kept for 72 h of cold stratification at 4 °C in dark. The plates were kept vertically at 90 °and grown under four different WL intensities (Long day, 16 h light/8 h dark) in the range of 150, 112, 75 to 38 μmol m ^−^ ^2^ s ^−^ ^1^ light in Percival light cabinets (Model No. CU36L6) at 22 °C with relative humidity of ~ 70%. Root phenotyping and RNA extraction were done with 6-days old and 5-days old seedlings respectively. Sample harvesting was approximately done at ZT = 12. The germination induction resulted in uniform germination among all genetic lines tested and confirmed by light microscopy, after 2 days of transferring in light. The root growth comparison was performed in 6-days old seedlings.

### RNA extraction and microarray analysis

RNA was extracted from the root tissues of 5-days old WT seedlings grown under different light intensities (38, 75, 112 and 150 μmol m ^−^ ^2^ s ^− ^^1^) with TRIzol reagent (Thermofisher Scientific, Cat. No.15596026), following the manufacturer’s protocol. RNA samples were subjected to DNaseI treatment with TURBO DNA free kit (Thermofisher Scientific, Cat. No. AM1907). For microarray analysis, the RNA quality was analysed with the Bioanalyzer (Agilent Bioanalyzer 2100 system). cDNA was prepared from RNA samples with RevertAid H Minus First Strand cDNA Synthesis Kit (Thermofisher Scientific, Cat. No. K1631). Microarray analysis was performed following Affymetrix GeneChip® WT PLUS Reagent Kit Manual Target Preparation for GeneChip® Whole Transcript (WT) Expression Arrays. Microarray experiment was done at ILS laboratory, Gurgaon, Haryana, India. Two independent biological replicates for each condition were used for microarray experiment in addition to this, three technical replicates were utilized. DEGs obtained from microarray were analysed with One-Way Between-Subject ANOVA (unpaired) test. Gene Level Differential Expression Analysis was performed and genes having minimum FC of 1.2 with FDR < 0.05 were selected for further analysis. The DEGs were categorized in six comparative light intensities such 150 vs 112 μmol m ^−^ ^2^ s ^− ^^1^ light, 150 vs 75 μmol m ^−^ ^2^ s ^−^ ^1^ light, 150 vs 38 μmol m ^−^ ^2^ s ^−^ ^1^ light, 112 vs 75 μmol m ^−^ ^2^ s ^− ^^1^ light, 112 vs 38 μmol m ^−^ ^2^ s ^−^ ^1^ light and 75 vs 38 μmol m ^−^ ^2^ s ^−^ ^1^ light.

### Venn diagram, gene enrichment (GO) pathway analysis and KEGG analysis

Venn diagrams were prepared for overlapping genes considering a combination of three comparative light conditions at a time. Gene lists from the microarray data were sorted and used for plotting Venn diagram using a custom script for R [67] using packages gdata [68], Vennerable [69], gplots [70], RBGL [71] and graph [72]. The script is available online on GitHub https://github.com/debadutta-patra/Venn-Diagram. GO pathway analysis was performed using Panther Over-representation test and the annotation version is GO Ontology database. The FISHER test was used and the categorization of DEGs under different GO terms was done considering FDR < 0.05. The KEGG search and colour pathway analysis was performed considering only a small group of DEGs.

### qRT-PCR analysis for DEGs

For quantitative qRT-PCR analysis, an aliquot of the cDNA samples was prepared from the same RNA used for microarray of WT. For validation of transcriptome data, qRT-PCR was performed with Ler, *35S::PhyBGFP* and *phyB-5* root samples*.* The qRT-PCR was performed using Biorad Evagreen kit (Bio-Rad SSoFast EvaGreen Supermix, Cat. No. 172–5203) and Bio-Rad C_1000_ Touch™ thermal cycler was used for qRT-PCR (CFX384™ Real Time System). The total volume of the reaction was 10 μl with 25 ng cDNA for WT, 50 ng cDNA for Ler, *35S::PhyBGFP* and *phyB-5* and each primer of 5 μM concentration was used. The software used for data analysis was Bio-Rad CFX Manager 3.81, the gene expression was analysed with FC of 1.2 and *p*-value < 0.05. The transcript level was normalized with a pair of primer specific for *POLYUBIQ* gene*.* qRT-PCR was done at least in triplicate and the data shown represent the mean + SE.

The forward and reverse primers are listed in the supplementary data (Additional file 6).

### Measurement and statistical analysis

The plates showing root phenotype were scanned at 600 dpi using HP Scanjet G4010 Flatbed Scanner. The lateral root growth pictures were captured with Nikon stereo zoom microscope (Nikon SMZ745T). The lateral root and adventitious root number were quantified under the stereo zoom microscope. The scanned images were used for measuring primary root length using ImageJ [73]. Mean comparison was done by one-way ANOVA with Tukey test for multiple mean comparison and with a probability threshold of 0.05. All the statistical analysis and graphs plotting were done using OriginPro (OriginLab, Northampton, MA).

## Additional files

Additional file 1: **Figure S1.** Detailed zoomed pictures of lateral root growth in 6-day old WT, *phyB-9* and *phyA-211* seedlings grown under WL intensities of 38, 75, 112 and 150 μmol m ^− 2^ s ^− 1^. Scale bar = 5 mm. Arrow head represents lateral roots. (PNG 1250 kb)

Additional file 2: **Figure S2.** Microscope images of lateral root growth for qualitative visualization in 6-day old WT, *phyB-9* and *phyA-211* seedlings grown under WL intensities of 38, 75, 112 and 150 μmol m ^− 2^ s ^− 1^. Scale bar = 5 mm. (PNG 4917 kb)

Additional file 3: List of DEGs Common in two, three, four or five comparative light conditions. (xlsx 1970 kb)

Additional file 4: List of Unique DEGs in specific comparative light conditions. (XLSX 40kb)

Additional file 5: **Figure S3.** Composite figure of GO analysis of DEGs under (a) 150 vs 112 μmol m ^− 2^ s ^− 1^ (b) DEGs under 150 vs 75 μmol m ^− 2^ s ^− 1^ (c) DEGs under 150 vs 38 μmol m ^− 2^ s ^− 1^ (d) DEGs under 112 vs 75 μmol m ^− 2^ s ^− 1^ (e) DEGs under 112 vs 38 μmol m ^− 2^ s ^− 1^ (f) DEGs under 75 vs 38 μmol m ^− 2^ s ^− 1^ light intensity. (PNG 40 kb)

Additional file 6: List of primers used for qRT-PCR. (DOCX 14 kb)

## Data Availability

The datasets which support the conclusion of this article have been included in the article and additional files have been provided separately.

## Abbreviations

*ACS8*: *ACC synthase 8*
*ARF18*: *Auxin response factor 18*
ARF2: Auxin response factor 2
*ARF4*: *Auxin response factor 4*
*ARR6*: *Type A response regulator 6*
*ATHB4*: *Arabidopsis thaliana homeobox protein 4*
BP: Biological Processes
CC: Cellular component
*CCA1*: *Circadian clock-associated 1*
*CIP1*: *COP1 interacting protein1*
*COL3*: *Constans like 3*
*COL9*: *Constans like 9*
*COP1*: *Constitutive photomorphogenesis 1*
CRY1: Cryptochrome 1
CRY2: Cryptochrome 2
*CSN6A*: *COP 9 signalosome complex subunit 6 A*
*CSN6B*: *COP 9 signalosome complex subunit 6 B*
*CYP81F2*: *Cytochrome P450 81F2*
DEGs: Differentially expressed genes
EPR1: Early phytochrome response 1
FC: Fold change
FDR: False Discovery Rate
*FHL*: *FHY1-like*
*FHY1*: *Far-red elongated hypocotyl 1*
GO: Gene ontology
*HFR1*: *Long hypocotyl in far-red light* 1
*HY5*: *Elongated hypocotyl 5*
KEGG: Kyoto Encyclopedia of Genes and Genomes
*KMD1*: *Kiss me deadly 1*
*LAX2*: *Like aux 2*
*LHY1*: *Late elongated hypocotyl 1*
MF: Molecular Function
*PAR2*: *Phytochrome rapidly regulated 2*
PHOT1: Phototropin 1
PHOT2: Phototropin 2
PHYA: Phytochrome A
PHYB: Phytochrome B
*PIF4*: *Phytochrome interacting factor 4*
*PRR9*: *Pseudo response regulator 9*
RIN: RNA Integrity No
*SAUR26*: *Small auxin upregulated RNA 26*
*SAUR9*: *Small auxin upregulated RNA 9*
SlCyp1: *Solanum lycopersicum* cyclophilin
*TAA1*: *Tryptophan aminotransferase of arabidopsis1*
*TOC1*: *Timing of cab expression 1*
UVR8: UVB-Resistance 8
WL: White light
