# Supplementary figures and images for: Uncovering the molecular signature underlying the light intensity-dependent root development in Arabidopsis thaliana
Source: BMC Genomics. 2019 Jul 20;20:596. doi: 10.1186/s12864-019-5933-5 (PMC6642530; doi:10.1186/s12864-019-5933-5)

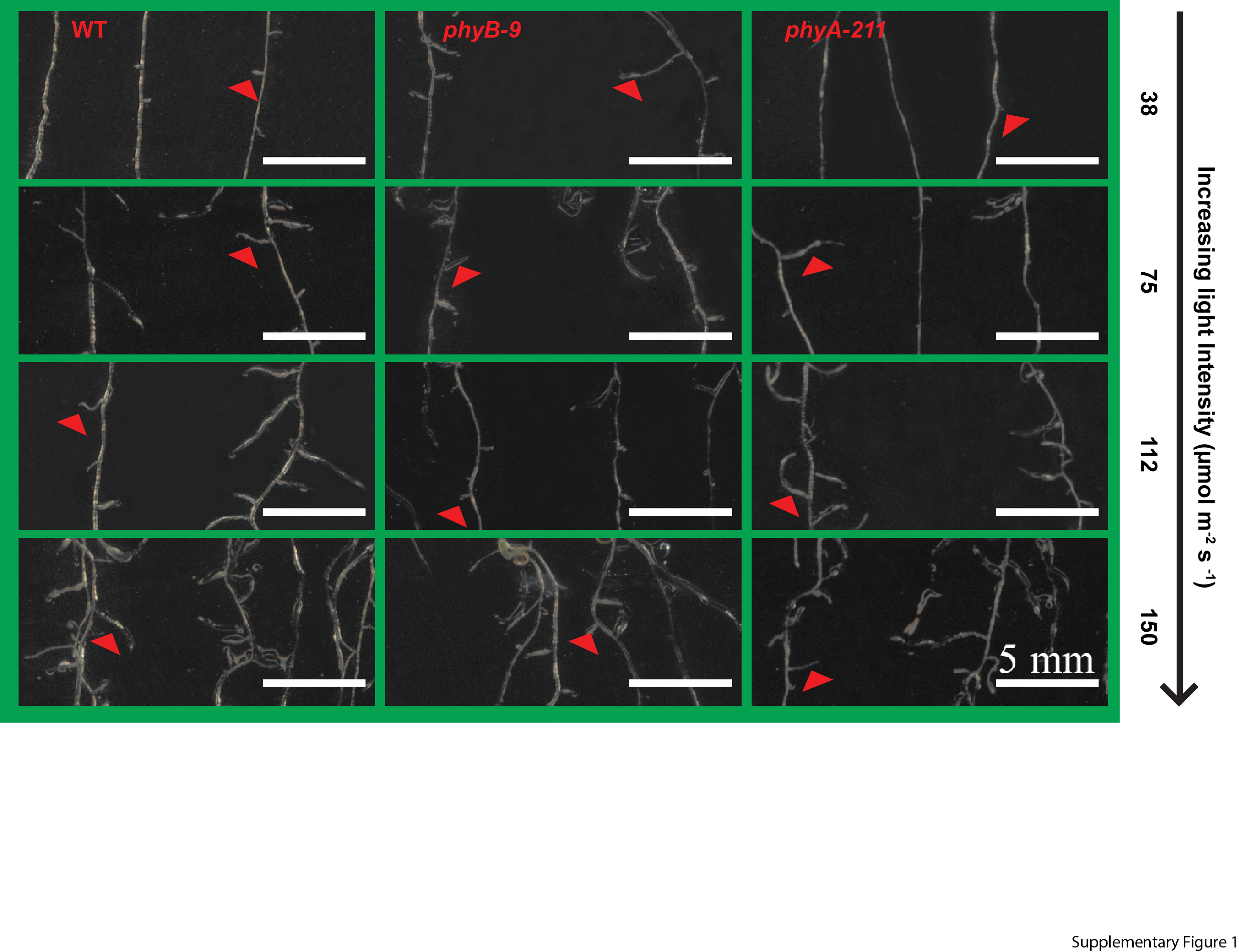

Supplement: Supplementary file 1 — Figure S1. Detailed zoomed pictures of lateral root growth in 6-day old WT, phyB-9 and phyA-211 seedlings grown under WL intensities of 38, 75, 112 and 150 μmol m − 2 s − 1. Scale bar = 5 mm. Arrow head represents lateral roots. (PNG 1250 kb) [file 12864_2019_5933_MOESM1_ESM.png]
