# Supplementary material for: Uncovering the molecular signature underlying the light intensity-dependent root development in Arabidopsis thaliana
Source: BMC Genomics. 2019 Jul 20;20:596. doi: 10.1186/s12864-019-5933-5 (PMC6642530; doi:10.1186/s12864-019-5933-5)
Supplement: Supplementary file 6 — List of primers used for qRT-PCR. (DOCX 14 kb) [file 12864_2019_5933_MOESM6_ESM.docx]

**Primers used for real-time PCR:**

| **Gene names** | **Forward primer (5’-3’)** | **Reverse primer (5’-3’)** |
| --- | --- | --- |
| **PAR2** | TCTCCTCCGTCTCCATCCTCCG | CATCTTCATCTTCTACGTCGGTCTCGC |
| **ARR6** | CCAATACCTTGGTTTGGATGTTGAGGA | GAGTTCATATCCAGTCATACCGGGC |
| **HY5** | ACGGCGAGTGCCGGAGTTTGGA | CCGGTGTCCTCCCTCGCTTC |
| **ARF18** | GGCGGAGCATGAAACAGATGAGG | CGAATGAAACTCTTGCTTAGTTGGTCC |
| **ARF2** | CGTGAACAGGGAAGACCATTCCAG | CAATTCCCTGCTTGTGAACCTTTGTGC |
| **ARF4** | TGACTGGAGTCTGTGACTTGGACCC | GTGTGGGAGAGAAACCGAGGGATC |
| **KMD1** | AGCTTCCTCCGATTCCTGGTCAAACC | ACCGAATCATGGGCCTGCCACG |
| **PIF4** | GCCAAAACCCGGTACAAAACCAGATC | GAGTCGCGGCCTGCATGTGTG |
| **IAA7** | AACTTTGGTGGAGGAGCAGCCGG | CCTGGTAGCTTTTGTACATCTTGAGGT |
| **EPR1** | ATCCTCGGAAATCGCCTGTTCCATATAC | GGTACACGAATTAGGCGAAGAGCATC |
| **COL3** | TGTTTCGACGAGAACGATAGATGTACC | GGTGATAACTGCACAGCTGGCGTC |
| **SAUR9** | GCTACTTCAACGACGTGCCAAAAGGT | AGCCACGAGATTGGGACCACATAGC |
| **SAUR26** | ATGGCTTTGGTGAGAAGTCTCTTTAGC | CTTCTTCTGGCTCTCGCCGACGTA |
| **LAX2** | ATGGAGAACGGTGAGAAAGCAGCTG | ACCAAGCATCATAAGCAGAGCCACC |
| **COL9** | ATGGTGTACTGTCGATCCGATGCAG | TTGCATCTCTCGCAGACAAGTGTCCG |
| **CIP1** | GCACGGGTGGAATCAGCAGAGG | GGTGCTTTCCGCCCGCTTGATC |
| **CSN6A** | CTCAGCTCAATCCTCCTGCTTCAATC | CTCTGGACACCGATCACACATCC |
| **CSN6B** | TCACGAATTGTACCAGCAACATGAACG | TCCTACCACCTCTTCGGCTGTG |
| **CCA1** | GGGAAGAGGGAAGTCAGAATAACAGG | GTCTGAGGTCCTTGCTCATTACCG |
| **TOC1** | TAGGTCCACCAACCCACAGA | TCAGCACCAAGACCACCATC |
| **PRR9** | CAGGTGAGCCAAAGACACCAACC | CGATATTCTCCTGGTTGCTGCTCG |
| **POLYUBIQ** | GGCCTTGTATAATCCCTGATGAA | AGAAGTTCGACTTGTCATTAGAAAGAAA |
